# Practical Lithium–Sulfur Batteries: An Integrated Design Roadmap from High‐Loading Cathodes to High‐Energy Pouch Cells

**DOI:** 10.1002/advs.75688

**Published:** 2026-05-19

**Authors:** Shihzad Shakil, Fan Wang, Lejun Fu, Yuye Huang, Hanhui Lei, Jiarui Huang, Terence Xiaoteng Liu

**Affiliations:** ^1^ Key Laboratory of Functional Molecular Solids of the Ministry of Education College of Chemistry and Materials Science Anhui Normal University Wuhu P. R. China; ^2^ School of Materials Science and Engineering Tongling University Tongling Anhui P. R. China; ^3^ School of Engineering Physics and Mathematics Faculty of Science and Environment, Northumbria University Newcastle upon Tyne UK

**Keywords:** data‐driven optimization, electrolyte‐cathode synergy, high‐loading electrodes, integrated system design, pouch cell validation, practical lithium–sulfur batteries

## Abstract

The transition to a sustainable energy future requires electrochemical storage systems that surpass conventional lithium‐ion batteries. The lithium–sulfur (Li–S) battery, with its high theoretical energy density and use of abundant sulfur, is a paramount contender for next‐generation applications. However, its commercialization is hindered by intrinsic challenges: the insulating nature of sulfur, the deleterious polysulfide shuttle effect, and severe volumetric expansion. For over a decade, research has focused on nanomaterial engineering under idealized laboratory conditions, yielding metrics often divorced from practical reality. This review argues that overcoming these barriers necessitates a decisive paradigm shift from isolated material breakthroughs to the synergistic integration of three interdependent frontiers. First, the scale‐up imperative, translating nanoscale innovations into manufacturable, high‐loading electrodes validated in lean‐electrolyte pouch cells (where the electrolyte‐to‐sulfur ratio, E/S is minimized). Second, electrolyte‐cathode synergy, co‐engineering an integrated system where components are mutually reinforcing. Third, systematic, data‐driven optimization using advanced operando characterization, multi‐physics modeling, and artificial intelligence (AI) to navigate the complex parameter space. By deconstructing these gaps, we synthesize a pragmatic roadmap emphasizing integration and practical validation. We posit that only through coordinated advances across these interconnected domains can the transformative potential of high‐energy‐density lithium–sulfur batteries be fully realized for applications like electric aviation and grid storage.

## Graphical abstract

This review presents an integrated design roadmap for practical lithium–sulfur batteries, moving beyond isolated material breakthroughs to the synergistic combination of three interdependent frontiers: Scale‐Up, Electrolyte‐Cathode Synergy, and AI/ML Data‐Driven Design. Key performance targets are highlighted alongside a staged roadmap to commercialization. This comprehensive graphical abstract encapsulates the entire scope of the review, from fundamental electrochemistry to manufacturing and data‐driven optimization.

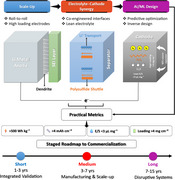

## 1 Introduction

The global transition to renewable energy and electric mobility is fundamentally constrained by the limitations of current electrochemical storage. While lithium‐ion (Li‐ion) batteries dominate the landscape, their theoretical energy density ceiling (∼350 Wh kg^−1^ at the cell level) is being approached, insufficient for the next generation of long‐range electric vehicles, aviation, and grid storage. This imperative for high‐energy‐density storage has catalyzed the search for “beyond Li‐ion” chemistries, among which the lithium–sulfur (Li–S) battery stands as a paramount contender. Operating on the conversion reaction between sulfur and lithium (S_8_ + 16Li^+^ + 16e^−^ ↔ 8Li_2_S), the Li–S system boasts a formidable theoretical specific energy of ∼2600 Wh kg^−1^ and a specific capacity of 1675 mAh g^−1^, significantly surpassing incumbent technology. Furthermore, it leverages sulfur an abundant, low‐cost, and environmentally benign material offering a compelling pathway to sustainable energy storage. This high energy density has been exploited in primary (non‐rechargeable) cells [1].

However, the journey from theoretical promise to practical application is obstructed by a triad of intrinsic challenges rooted in the fundamental electrochemistry of the sulfur cathode. First, the insulating nature of elemental sulfur (S_8_) and the final discharge product, lithium sulfide (Li_2_S), necessitates intimate integration with conductive hosts to enable electron transfer, a requirement that becomes acute under practical high‐mass‐loading conditions. Second, the polysulfide shuttle effect arises from the dissolution of intermediate lithium polysulfide species (Li_2_S_x_, 4 ≤ x ≤ 8) in conventional ether‐based electrolytes. These soluble species diffuse between the electrodes, leading to irreversible active material loss, parasitic reactions at the lithium anode, rapid capacity fade, and low Coulombic efficiency. Third, the pronounced volume change (∼80%) between S_8_ and Li_2_S induces severe mechanical stress, pulverizing cathode structures and destabilizing the electrode‐electrolyte interface over extended cycling [2, 3].

**SCHEME 1:**
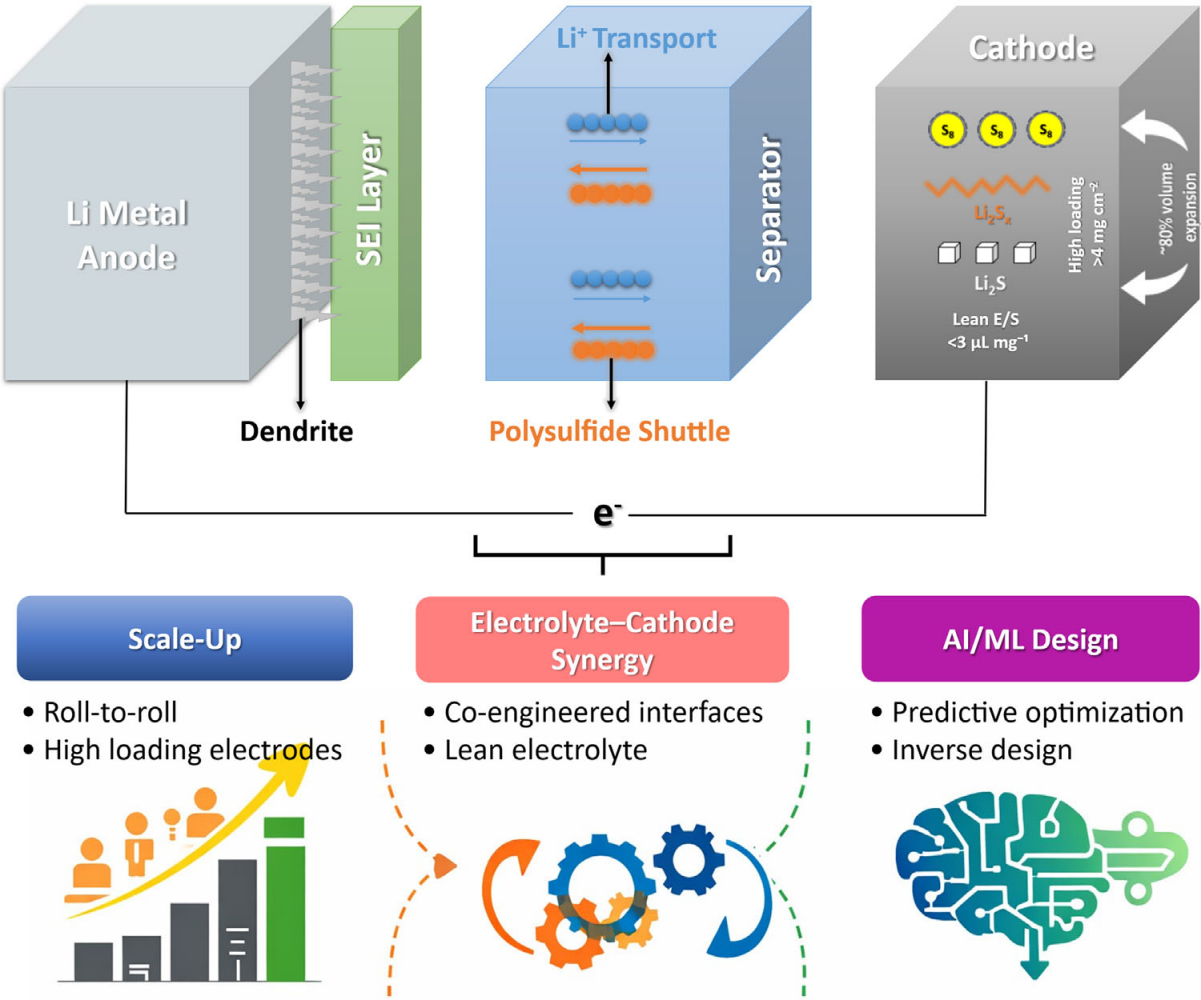
Integrated schematic illustrating the multiscale challenges and design strategies for practical lithium–sulfur batteries. The diagram links nanoscale sulfur redox chemistry (S_8_ → Li_2_S_X_ → Li_2_S), electrode‐level porous host design with high sulfur loading, and device‐level pouch cell architecture. Key physical, chemical, and system challenges are highlighted, while three integrated research frontiers scale‐up, electrolyte–cathode synergy, and AI/ML‐driven design are presented as the pathway toward practical high‐energy‐density Li–S batteries. Original synthesis by authors.

Compounding these issues is the challenge of maintaining sufficient ionic conductivity within thick, high‐loading electrodes under lean electrolyte conditions, where restricted lithium‐ion transport can lead to concentration polarization, incomplete sulfur utilization, and premature cell failure. This ionic transport limitation becomes increasingly severe as electrodes are thickened to achieve commercially relevant areal capacities and deserves equal attention alongside the more commonly cited electronic conductivity concerns. These interrelated challenges including poor electronic conductivity, polysulfide shuttling, volume expansion, ion transport limitations, and system‐level constraints collectively hinder the translation of sulfur cathodes from laboratory‐scale coin cells to practical pouch‐cell configurations, as schematically illustrated in Scheme 1.

The evolution of Li–S research reflects a concerted effort to confront these challenges. The initial decade was dominated by proof‐of‐concept nanostructuring strategies, primarily focused on encapsulating sulfur within highly conductive, porous carbon matrices (e.g., mesoporous carbon, carbon nanotubes, graphene). This approach successfully mitigated the insulating issue and physically confined polysulfides, leading to impressive performance in idealized laboratory settings [4, 5]. However, this phase often relied on low sulfur loadings (<2 mg cm^−2^), excessive electrolyte volumes (electrolyte‐to‐sulfur ratio, E/S > 15 µL mg^−1^), and thin electrodes, metrics divorced from commercial reality. This realization sparked a critical paradigm shift in the field: the recognition of practical metrics. Research focus is now judged against parameters essential for high energy density at the cell level: high sulfur loading (>4 mg cm^−2^), lean electrolyte conditions (E/S < 3 µL mg^−1^), and high cathode areal capacity (>4 mAh cm^−2^). Performance in coin cells under these stringent conditions, and ultimately in multilayer pouch cells, has become the true benchmark of progress [6].

To contextualize these metrics, it is instructive to define the quantitative boundary conditions required for a practical Li–S pouch cell to achieve a commercially relevant energy density, for instance, 500 Wh kg^−1^ at the cell level [7, 8]. Such a target imposes tightly coupled constraints on multiple parameters [9]. First, the sulfur areal loading must typically exceed 4–5 mg cm^−2^ to provide sufficient active material per unit area [8, 10]. Second, the electrolyte‐to‐sulfur (E/S) ratio must be ruthlessly minimized to below 3 µL mg^−1^; excess electrolyte dilutes energy density by adding inactive mass without contributing to capacity [11]. Third, the sulfur content in the cathode composite should be above 60–70 wt.% to minimize the mass fraction of inactive hosts and binders. Fourth, the negative‐to‐positive capacity ratio (N/P ratio) must be kept low, ideally below 2, to avoid excessive lithium metal that would otherwise reduce cell‐level energy density and pose safety risks [10]. Fifth, the cathode porosity must be optimized typically between 40–60% to accommodate volume expansion while maintaining ionic transport and electronic percolation [9]. Finally, these parameters must collectively yield a stable areal capacity exceeding 4–5 mAh cm^−2^ over hundreds of cycles [7, 10]. These boundary conditions, derived from rigorous cell‐level energy density calculations, form the quantitative framework against which the strategies discussed in this review must be evaluated.

### 1.1 Scope and Positioning of This Review

The rapid expansion of lithium–sulfur battery research has generated a wealth of review articles addressing specific facets of the field, from cathode design and electrolyte engineering to mechanistic investigations and emerging characterization techniques. While these contributions have advanced our understanding, a comprehensive, system‐level framework that integrates the interdependent challenges of practical deployment remains conspicuously absent. This section positions the present review within the contemporary literature, highlighting its unique contributions across four key dimensions: scope, metrics, framework, and conclusions. Table 1 compares the present work with eight representative reviews published in 2024–2025, each selected for its distinct focus and authoritative source. The comparison illustrates the landscape of current Li–S reviews and underscores the novel integrative perspective offered herein.

**TABLE 1:** Comparison of the present review with representative recent reviews on lithium–sulfur batteries.

Review	Focus area	Metrics emphasis	Framework type	Key contribution
Li et al. [12]	Microenvironment engineering (structural, lithiophilicity, sulfiphilicity)	Moderate–discusses performance enhancements	Microenvironment‐centric	Introduces a novel “microenvironment engineering” paradigm for rational design
Sawangphruk [13]	Broad materials coverage (MOFs, COFs, MXenes, solid‐state electrolytes)	Moderate–surveys reported capacities and cycling stability	Material‐centric, categorized by material class	Comprehensive overview of emerging materials with future research directions
Wu et al. [14]	Metal‐based composite sulfur cathodes	Moderate–includes quantitative analysis of sulfur loading and content	Quantitative area‐proportional analysis	Systematically categorizes research areas and identifies most studied strategies
Ye et al. [15]	All‐solid‐state Li–S batteries	High–provides detailed analysis of specific energy, sulfur loading, and content	Parameter‐centric	Quantifies critical design parameters for solid‐state cells and offers fabrication guidelines
He et al. [16]	Polymer‐based solid‐state Li–S batteries	Moderate–reviews electrode fabrication, anode protection, electrolyte formulation	Component‐centric (cathode, electrolyte, anode, interface)	Comprehensive coverage of challenges and strategies in polymer‐based systems
Yari et al. [3]	Data‐driven benchmarking of functional sulfur hosts	Very high–analyzes 866 galvanostatic plots to link design parameters with specific energy	Statistical/data‐centric	First largescale statistical benchmarking; identifies optimal design regions
Offermann et al. [17]	Fast‐charging Li–S batteries	High–focuses on rate capability (≥2 C) and associated metrics	Performance‐centric (rate)	Dedicated analysis of fast‐charging mechanisms and electrode/electrolyte requirements
This Review	Integrated system design from cathode to pouch cell	High–consistently applies 500 Wh kg^−1^ boundary conditions throughout	Three interdependent frontiers (scaleup, electrolyte–cathode synergy, data‐driven optimization)	First review to unify manufacturing scalability, co‐engineering principles, and AI/ML‐driven discovery into a single actionable roadmap

As Table 1 illustrates, existing reviews typically adopt a material‐, component‐, or application‐centric lens, often with moderate or high emphasis on performance metrics but rarely integrating the multiple frontiers that must advance simultaneously for commercial success. The present review fills this gap by offering a tripartite framework that explicitly links scalable manufacturing, electrolyte–cathode co‐engineering, and data‐driven optimization. Moreover, it maintains a rigorous focus on the quantitative boundary conditions for achieving 500 Wh kg^−1^ at the pouch cell level, ensuring that every discussed strategy is evaluated against practical viability. Finally, it culminates in a staged roadmap with measurable milestones, providing concrete guidance for researchers and clear benchmarks for progress. This integrative, system‐level perspective distinguishes the present work and positions it as a timely and valuable contribution to the Li–S community.

## 2 From Coin Cell to Pouch Cell: The Scaling‐Up Imperative

**FIGURE 1:**
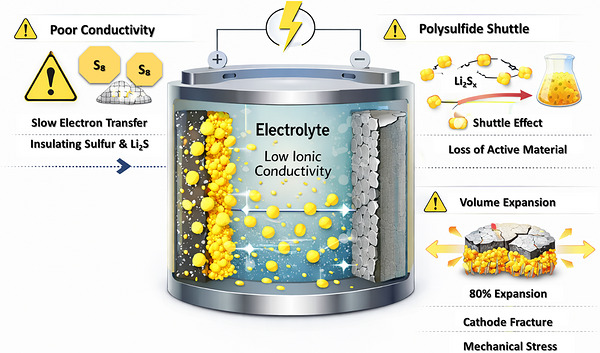
Schematic illustration of the key challenges in conventional lithium–sulfur batteries: poor electronic conductivity of sulfur and Li_2_S, low ionic conductivity in the electrolyte, polysulfide shuttle effect leading to active material loss, and volume expansion‐induced cathode fracture. These interrelated issues collectively impede the translation of laboratory breakthroughs to practical pouch cell performance. Original synthesis by authors.

Under the stringent constraints of high sulfur loading and lean electrolyte, the design requirements for cathode architectures undergo a critical paradigm shift. Strategies that succeed in flooded, low‐loading coin cells often fail under practical conditions, exposing fundamental trade‐offs in conductivity, polysulfide management, and mechanical stability. The evolution of cathode design reflects a continuous refinement toward multifunctional, integrated systems capable of operating in this harsh regime. This progression can be traced from early confinement strategies to advanced catalytic and topologically engineered hosts [18]. The multifaceted challenges that emerge under practical operating conditions are schematically illustrated in Figure 1. These interconnected failure modes including polysulfide shuttle, electrolyte depletion, ionic transport limitations, and lithium anode degradation must be addressed simultaneously to achieve commercially viable pouch cell performance.

### 2.1 The Disconnect in Performance Metrics

At the heart of the scaling challenge lies a profound disconnect in the key parameters that define performance between fundamental academic research and the demands of practical application. For lithium–sulfur batteries, three metrics are paramount: sulfur loading (mg_s_ cm^−2^), which determines the total active material per unit area; the electrolyte‐to‐sulfur ratio (E/S, µL mg^−1^), which governs ion transport and the concentration of deleterious polysulfides; and the resulting cathode areal capacity (mAh cm^−2^), the direct determinant of cell‐level energy density [19]. The typical academic pursuit has long operated within a forgiving, electrolyte‐flooded regime: sulfur loadings below 2 mg cm^−2^, E/S ratios often exceeding 15 µL mg^−1^, and consequently, modest areal capacities under 3 mAh cm^−2^. While this regime is excellent for probing fundamental electrochemistry and material concepts, it masks critical failure modes. The excess electrolyte dilutes polysulfide concentration, minimizing shuttle effects and side reactions, while thin electrodes circumvent issues of ionic/electronic resistance and mechanical stress. In stark contrast, commercial viability demands a harsh, lean‐electrolyte paradigm. To achieve cell‐level energy densities surpassing 500 Wh kg^−1^, practical cells require sulfur loadings above 4–5 mg cm^−2^, E/S ratios ruthlessly minimized to below 3 µL mg^−1^, and areal capacities consistently exceeding 4–5 mAh cm^−2^.

This target condition creates a challenging environment of high polysulfide concentration, restricted lithium‐ion flux, and severe volumetric strain [20, 21, 22]. Consequently, a cathode material exhibiting superb cyclability at 1 mg cm^−2^ and E/S = 20 µL mg^−1^ may fail catastrophically under practical parameters, rendering initial performance data a misleading indicator of real‐world potential. A rigorous framework for understanding how these parameters dictate practical cell‐level energy density was established by Zhou et al. [23]. They formulated energy density equations based on these critical parameters, introducing descriptors (R_weight_, R_energy_) to quantify the mass‐ and energy‐level compromises inherent in moving from cathode material to full cell. This work crystallized the community's quantitative understanding of the ‘harsh, lean‐electrolyte paradigm’ required for commercial viability.

Recognizing this disconnect is therefore the essential first step in rational, application‐oriented cathode design [23]. Moving beyond phenomenological performance reporting, recent research has begun to establish fundamental design principles for catalysts that must function under these severe conditions. A pivotal study by Geng et al. [24] directly links atomic‐scale catalyst properties to performance in the lean‐electrolyte, high‐loading regime. They identified that strong polysulfide binding, a traditionally sought‐after property, can actually be detrimental as it disrupts the Electric Double Layer (EDL) at the catalyst‐electrolyte interface, leading to passivation and hindered mass transport failures that are acutely exposed under high polysulfide concentrations.

By introducing a competitive adsorption factor (f_sulfur_) and engineering a Co‐Zn alloy catalyst with *moderate* adsorption strength, they achieved a near‐theoretical platform capacity and exceptional anti‐passivation ability. Critically, this performance was validated under high sulfur loading and a lean E/S ratio of 5 µL mg^−1^, culminating in a pouch cell with an energy density of 464 Wh kg^−1^. This work provides a quantitative descriptor that directly guides the design of catalytic hosts for the “harsh paradigm,” illustrating how fundamental electrochemistry must be re‐examined through the lens of practical operating conditions [24].

Table 2 compiles representative high‐performance Li–S cells reported in recent literature, categorized by cell format. Several trends emerge from this compilation: First, the definition of “practical” metrics has sharpened considerably. For pouch cells targeting >400 Wh kg^−1^, the community now recognizes that sulfur loadings should exceed 4 mg cm^−2^, E/S ratios must be minimized below 5 µL mg^−1^ (and ideally <3 µL mg^−1^), and areal capacities should surpass 4 mAh cm^−2^. Cells meeting all three criteria simultaneously such as the Co‐PCL catalytic cathode [27] and pre‐lithiated 1T‐MoS_2_ host [26] represent the current state‐of‐the‐art. However, the limited cycle life of these demonstrations (e.g., 20 cycles for [27]) highlights the remaining gap between laboratory breakthroughs and commercial viability. Second, a persistent gap remains between coin cell demonstrations and pouch cell validation. While remarkable areal capacities (>20 mAh cm^−2^) have been achieved in coin cells under flooded conditions [29], these metrics rarely translate directly to pouch cells, where electrolyte distribution, stack pressure uniformity, and current collection become critical variables. Notably, the laser‐printed pouch cell [28] demonstrates that innovative fabrication techniques can bridge this gap, achieving high loadings with lean electrolyte.

**TABLE 2:** Recent performance benchmarks in Li–S battery research.

Category	Battery system	Sulfur loading (mg cm^−2^)	Sulfur content (wt%)	Active material ratio (S:Host:Binder)	Electrolyte composition	E/S ratio (µL mg^−1^)	Areal capacity (mAh cm^−2^)	Cycle life (cycles @ retention)	Key performance metric	References
Pouch cells	Flexible Li–S pouch cell (All‐fibrous, CNT‐based)	8.5‐10.4	∼70%	70:25:5	1 M LiTFSI + 0.25 M LiNO_3_ in DOL/DME	15	8.4 @ 0.1C	Not reported	443 Wh kg^−1^, mechanical flexibility	[25]
	Li–S pouch cell (Pre‐lithiated 1T‐MoS_2_ host)	>4	65%	65:30:5	1 M LiTFSI in DOL/DME with LiNO_3_	≤5	High (inferred)	200 @ 85.2%	441 Wh kg^−1^, 735 Wh L^−1^	[26]
	Li–S pouch cell (Co‐PCL Catalytic cathode)	3.4 (pouch)	70%	70:20:10	LHCE (LiFSI in DME/DOL with diluent)	2.4	∼9.3 (calc.)	20 @ ∼90%	507 Wh kg^−1^, lean E/S	[27]
	Li–S pouch cell (Laser‐printed MnOx‐Hal‐S@CF)	7.2 (per side)	60%	60:35:5	1 M LiTFSI + 0.2 M LiNO_3_ in DOL/DME	5	∼7.8	Not reported	Single‐step fabrication	[28]
Coin cells (High loading)	Li–S coin cell (CNTs@TiN‐TiO_2_ sponge)	15.0	75%	75:20:5	1 M LiTFSI + 0.2 M LiNO_3_ in DOL/DME	10	21.5	500 @ 85% (2C)	Record‐high areal capacity	[29]
	Li–S coin cell (γS‐CNFs in carbonate electrolyte)	5.0	60%	60:35:5	carbonate electrolyte (e.g., EC/DEC with LiPF_6_)	20	∼2.35 @ 0.1C	>4000 @ 0.04% decay/cycle	Ultra‐long life in carbonate	[30]
	Li–S coin cell (Saccharide‐based binder)	10.5	70%	70:20:10	1 M LiTFSI + 0.2 M LiNO_3_ in DOL/DME	∼8.6–22	∼5.1 @ 0.2C	1000 @ ∼700 mAh g^−1^ retained	97% S utilization	[31]
All‐solid‐state	All‐solid‐state Li–S (C_3_N_4_/N‐Graphene host)	∼11.3	N/A	Composite with solid electrolyte	Li_6_PS_5_Cl solid electrolyte	Solid	11.3 @ 60°C	Stable @ 2 mAh cm^−2^ (RT)	High areal capacity in ASSB	[32]
	All‐solid‐state Li–S (ACSV cathode composite)	>2.0 (Li_2_S‐based)	N/A	Composite with solid electrolyte	Li_3_PS_4_ solid electrolyte	Solid	>2.0	100 @ 82.8%	1009 mAh g^−1^ @ 0.05C	[33]

Third, alternative paradigms are emerging that bypass traditional trade‐offs. All‐solid‐state Li–S batteries [33] eliminate polysulfide dissolution entirely but introduce interfacial challenges that limit room‐temperature performance. Carbonate‐compatible cathodes like γS‐CNFs [30] offer exceptional cycle life (>4000 cycles) by avoiding polysulfide formation, though at the cost of lower areal capacity. Fourth, reporting standards remain inconsistent. Critical parameters such as sulfur content in the composite, active material ratios, and detailed electrolyte compositions are often omitted, making cross‐study comparisons difficult. We advocate for standardized reporting that includes all columns shown in Table 1, with particular emphasis on simultaneous fulfillment of high loading (>4 mg cm^−2^), lean E/S (<5 µL mg^−1^), and high areal capacity (>4 mAh cm^−2^) in pouch cell formats. Papers reporting only coin cell data under flooded conditions should be viewed as preliminary material screens rather than demonstrations of practical viability a distinction we emphasize throughout this review.

#### 2.1.1 Explicit Differentiation of Failure Mechanisms: Coin Cells vs. Pouch Cells

While the preceding section established the quantitative gap in performance metrics between idealized coin cells and practical pouch cells, a deeper mechanistic understanding of *why* these gaps exist is essential for rational design. The failure modes that dominate in small‐scale, flooded coin cells differ fundamentally from those that dictate the lifespan of large‐format, lean‐electrolyte pouch cells. Recognizing these divergent failure paradigms is critical for translating material innovations from laboratory breakthroughs to commercial realities [34]. In conventional coin cells operated under flooded electrolyte conditions (E/S > 15 µL mg^−1^), the primary failure mechanism is the well‐documented polysulfide shuttle effect. The large volume of electrolyte readily dissolves the long‐chain lithium polysulfides (Li_2_S_x_, 4 ≤ x ≤ 8) generated during cycling, creating a high concentration of soluble species that migrate freely between the electrodes. This continuous dissolution, migration, and parasitic reaction at the lithium anode leads to irreversible active material loss, corrosion of the lithium metal, and gradual capacity fade over extended cycling. However, the flooded electrolyte acts as a buffer, diluting polysulfide concentrations and delaying the onset of catastrophic failure. In this regime, the electrolyte volume is not a limiting factor, and thin electrodes (<2 mg cm^−2^ sulfur loading) minimize ionic/electronic transport resistances, allowing the shuttle effect to manifest as the dominant, albeit slow, degradation pathway.

In stark contrast, practical pouch cells operating under stringent lean‐electrolyte conditions (E/S < 3 µL mg^−1^) and high sulfur loadings (>4 mg cm^−2^) experience a fundamentally different set of failure modes. The failure paradigm shifts from polysulfide management to transport starvation, localized polarization, and irreversible electrolyte depletion. The limited electrolyte volume cannot accommodate extensive polysulfide dissolution; instead, the electrolyte itself becomes a consumable component. Parasitic reactions at both electrodes including continuous solid‐electrolyte interphase (SEI) formation on the lithium anode and electrolyte decomposition at the cathode progressively consume the scarce electrolyte. This leads to progressive dry‐out, increased internal resistance, and ultimately sudden cell death [35]. Systematic failure analyses of high‐energy‐density Li–S pouch cells have confirmed electrolyte exhaustion as the key limiting factor. Chen et al. demonstrated that failed 400 Wh kg^−1^ pouch cells retained structurally intact cathodes and anodes with considerable residual capacity; reinjecting fresh electrolyte recovered the discharge capacity, conclusively identifying electrolyte depletion as the primary failure cause [36]. Similarly, Cheng et al. showed that 700 Wh kg^−1^‐level pouch cells failed after only three cycles due to rapidly increased polarization at the second discharge plateau, with failure analysis again pointing to electrolyte exhaustion [21].

Compounding electrolyte depletion are severe transport limitations inherent to thick, high‐loading electrodes. Under lean electrolyte, the porous cathode network suffers from restricted lithium‐ion diffusion, leading to steep concentration gradients and incomplete sulfur utilization, particularly in regions far from the separator. This localized polarization is exacerbated by pore blockage, where insoluble discharge products (Li_2_S) precipitate within the porous architecture, progressively obstructing ion transport pathways and increasing tortuosity. Furthermore, current distribution inhomogeneities across large‐area pouch cell electrodesabsent in small coin cells create localized overpotentials and non‐uniform sulfur utilization, accelerating failure. The lithium metal anode also experiences amplified degradation in pouch cells. While polysulfide corrosion remains an issue, the dominant failure mode shifts to mechanical degradation and interfacial instability driven by non‐uniform stack pressure and large volumetric changes. In operando pressure studies have revealed that the lithium anode dominates the thickness variation of the entire pouch cell, with cumulative stress causing progressive delamination, cracking, and loss of electrical contact [37, 38].

Recent work has further elucidated that the cathodic kinetics become severely impeded under lean‐electrolyte conditions. Zhao et al. demonstrated through polarization decoupling analysis that activation polarization rather than concentration or ohmic polarizationconstitutes the main challenge in lean‐electrolyte cells, as the sluggish interfacial charge transfer at the cathode becomes rate‐limiting when electrolyte volume is minimized. This finding underscores that electrocatalysts designed to accelerate sulfur redox kinetics are even more effective in lean‐electrolyte pouch cells than in flooded coin cells, directly addressing the shifted failure paradigm [39]. In summary, the failure mechanism of Li–S batteries undergoes a fundamental transformation when scaling from flooded coin cells to lean pouch cells. While the polysulfide shuttle remains a concern, it is superseded by the more immediate and catastrophic triad of electrolyte exhaustion, transport starvation, and activation polarization under practical conditions. Any material or cell design strategy destined for commercial relevance must therefore be evaluated against these shifted failure paradigms, not merely against shuttle mitigation in flooded cells.

### 2.2 Cathode Architectures under Practical Constraints: Functional Classification and Design Strategies

Under the stringent constraints of high sulfur loading and lean electrolyte, the design requirements for cathode architectures undergo a critical paradigm shift. Strategies that succeed in flooded, low‐loading coin cells often fail under practical conditions, exposing fundamental trade‐offs in conductivity, polysulfide management, and mechanical stability. The evolution of cathode design reflects a continuous refinement toward multifunctional, integrated systems capable of operating in this harsh regime, progressing from early confinement strategies to advanced catalytic and topologically engineered hosts [18]. To translate cathode innovations from coin cells to practical pouch cells, it is essential to understand how different material architectures address the specific failure modes that dominate under lean‐electrolyte, high‐loading conditions. Rather than cataloging materials chronologically, this section synthesizes the literature by functional mechanism physical confinement, chemical adsorption, catalytic conversion, and transport enhancement and explicitly links each mechanism to the mitigation of concentration polarization, reaction heterogeneity, and transport starvation in practical pouch cells.

#### 2.2.1 Physical Confinement Hosts

Early lithium–sulfur research was dominated by efforts to physically confine sulfur within conductive, porous matrices to address its insulating nature. Porous carbons including mesoporous carbon, carbon nanotubes, graphene, and sustainable derivatives from biomass such as shaddock peel served as foundational hosts, demonstrating improved conductivity and cycle life in idealized laboratory settings [40, 41]. Efforts to perfect this approach led to highly integrated nanocomposites like three‐dimensional porous graphitic carbon with uniformly distributed sulfur nanoparticles, which achieved very high sulfur content and highlighted the benefits of nanoscale sulfur distribution within a conductive network [42]. While effective at improving electronic conductivity and providing initial polysulfide retention, purely physical confinement hosts are often overwhelmed under the high local polysulfide concentrations found in thick, practical electrodes, necessitating additional chemical or catalytic functionality.

#### 2.2.2 Chemical Adsorption Mediators

**FIGURE 2:**
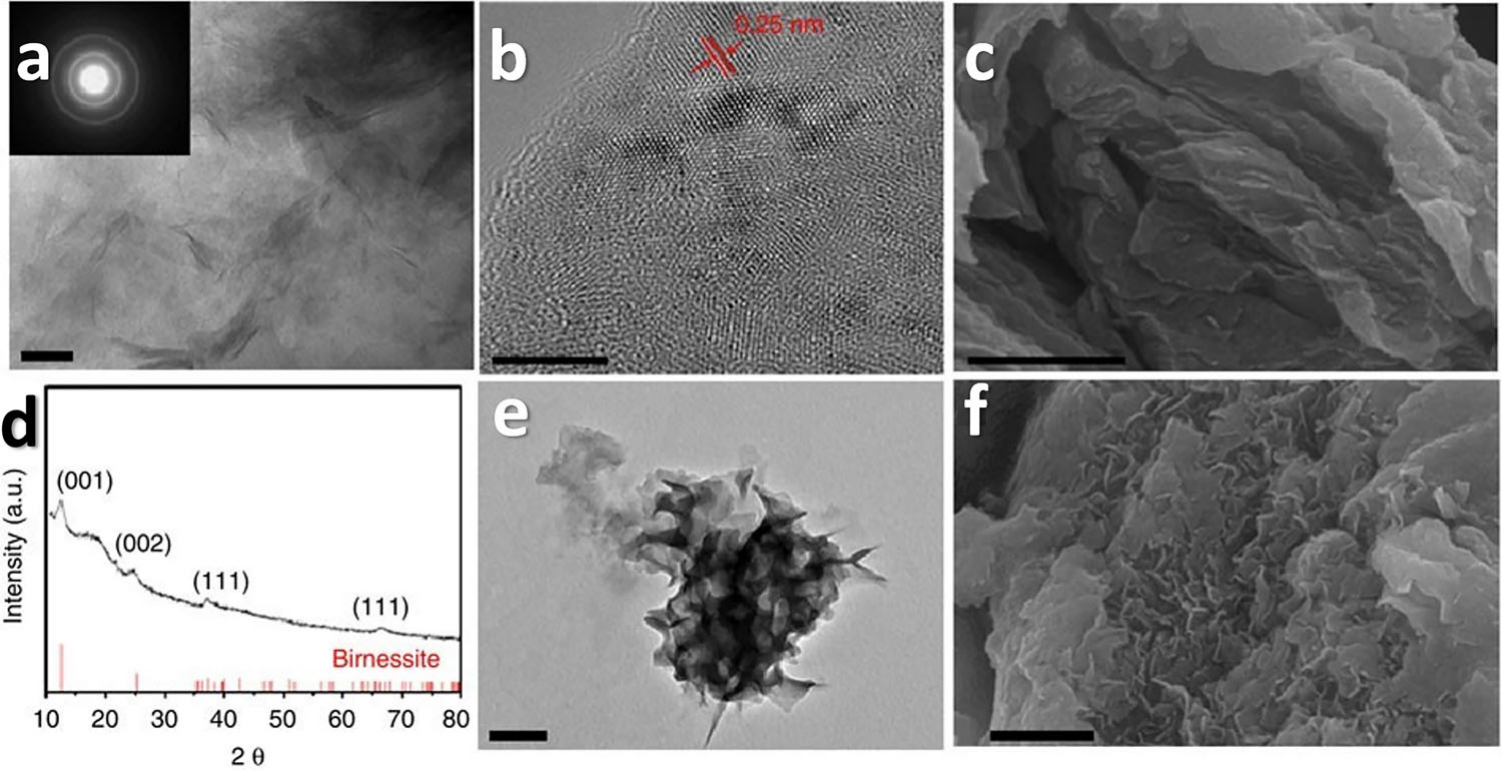
(a) TEM image of MnO_2_ nanosheets and its corresponding selected area electron diffraction pattern. (b) High‐resolution TEM image of MnO_2_ nanosheets. (c) SEM image of the MnO_2_ nanosheets. (d) XRD of MnO_2_ nanosheets. (e) TEM and (f) SEM images of the S/MnO_2_ nanosheets composite [43]. Adapted with permission from Ref. [43]. Copyright 2015, Springer Nature.

The inherent limitation of non‐polar carbons is their weak, physical‐only interaction with polysulfides, a mechanism easily overwhelmed under the high local concentrations found in thick, practical electrodes. This recognition sparked a pivotal shift from physical confinement to chemical mediation. Seminal work using manganese dioxide nanosheets revealed that MnO_2_ does not merely adsorb polysulfides but chemically reacts with them to form surface‐bound intermediates that catalytically promote conversion to Li_2_S, establishing chemical mediation as a superior design principle over simple physical containment [43]. The structural characterization of this material is presented in Figure 2. Specifically, Figure 2a shows a transmission electron micrscopy (TEM) image of MnO_2_ nanosheets with its corresponding selected area electron diffraction pattern (inset), confirming the crystalline nature of the nanosheets. Figure 2b provides a high‐resolution TEM image revealing the detailed lattice structure of an individual MnO_2_ nanosheet. Figure 2c displays an SEM image showing the overall morphology and uniform distribution of the MnO_2_ nanosheets. Figure 2d presents the X‐ray diffraction (XRD) pattern, which confirms the phase purity and crystal structure (birnessite phase) of the synthesized MnO_2_. Finally, Figure 2e,f shows TEM and scanning electron microscopy (SEM) images, respectively, of the S/MnO_2_ nanosheets composite, demonstrating the uniform distribution of sulfur within the MnO_2_ host matrix. This inspired the exploration of other polar host materials. For instance, the design of titanium monoxide@carbon hollow spheres combined the metallic conductivity of a TiO core with a polar surface for strong polysulfide adsorption, underscoring the ideal of integrating electrical conductivity with surface polarity [44].

The exploration of polar materials extended to various metal oxides like TiO_2_, where strategies such as creating oxygen vacancies via hydrogen reduction were shown to enhance polysulfide adsorption and stability [45]. Beyond composition, the very atomic arrangement of a host material emerged as a powerful design lever. Amorphizing cobaltous oxide nanosheets was shown to drastically alter surface electronic states, leading to stronger polysulfide binding and demonstrating how deliberate manipulation of material disorder can unlock enhanced functionality [46]. However, a critical balance was identified: overly strong adsorption can poison catalytic sites. Foundational work elucidated a volcano‐shaped relationship between polysulfide adsorption strength and catalytic activity, establishing that optimal performance requires moderate adsorption that facilitates conversion without leading to passivation [47]. By chemically binding polysulfides, these polar hosts localize active material within the cathode, reducing the concentration of soluble species that can migrate and cause reaction heterogeneity. This is particularly critical in thick electrodes where diffusion path lengths are long and uniform sulfur utilization is challenging.

#### 2.2.3 Catalytic Conversion Promoters

**FIGURE 3:**
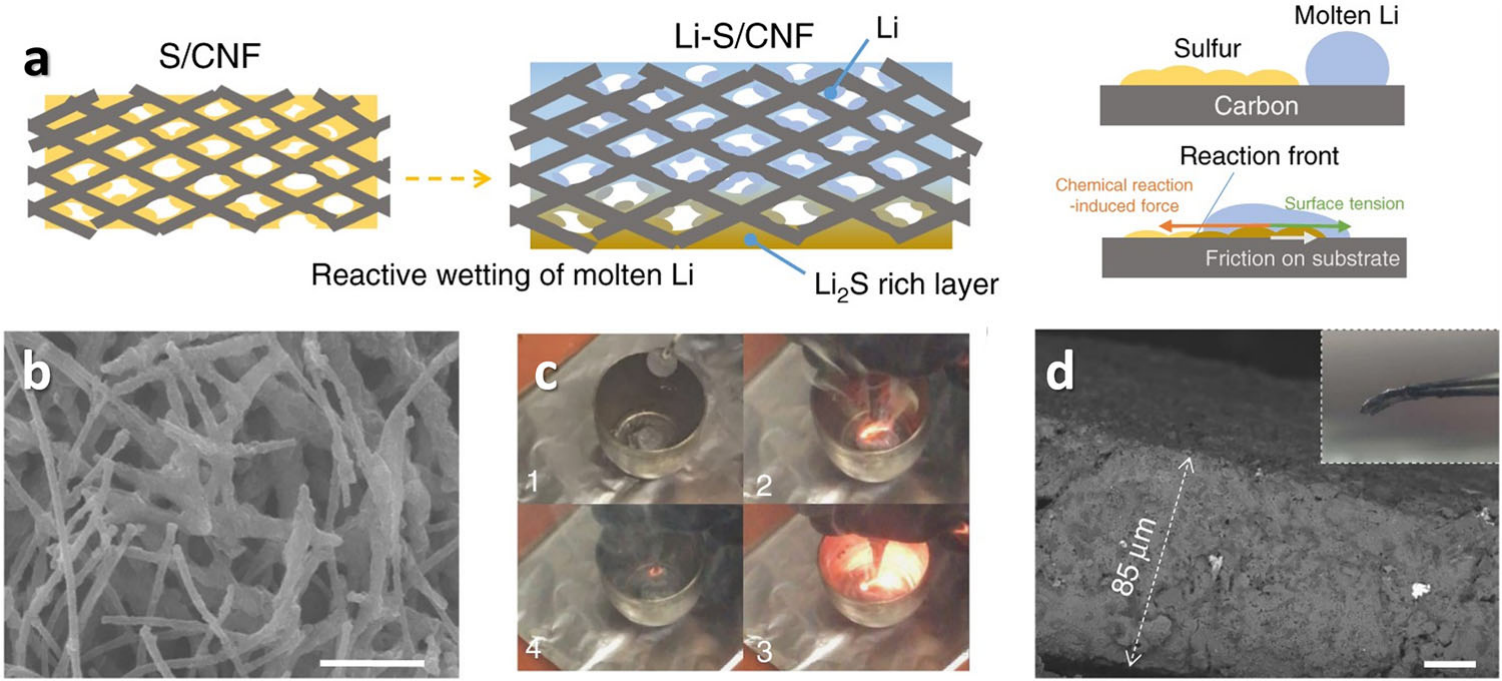
(a) Cartoons illustrating the fabrication process. (b) SEM of the surface of the pristine CNF. (c) Snapshots taken during the fabrication, showing the sparkling reaction between molten lithium and the sulfur‐impregnated CNF. (d) Cross sectional SEM of the Li/S‐CNF [48]. Adapted with permission from Ref. [48]. Copyright 2019, Springer Nature.

The logical progression from chemisorbing hosts was the deliberate design of hosts with intrinsic catalytic functionality to accelerate the sluggish kinetics of polysulfide conversion. This search extended to emerging materials such as black phosphorus quantum dots, which served as efficient, metal‐free electrocatalysts due to their abundant active edge sites and were integrated into conductive frameworks [48]. The fabrication process and resulting morphology of a representative carbon nanofiber (CNF)‐based host are illustrated in Figure 3. As, Figure 3a presents a schematic cartoon illustrating the fabrication process, where sulfur‐impregnated CNF reacts with molten lithium via reactive wetting, driven by chemical reaction‐induced forces and surface tension. Figure 3b shows an SEM image of the surface of the pristine CNF, revealing its interconnected fibrous network structure. Figure 3c provides snapshots taken during the fabrication process, showing the sparkling reaction between molten lithium and the sulfur‐impregnated CNF, where a Li_2_S‐rich layer forms at the reaction front. Figure 3d displays a cross sectional SEM image of the resulting Li/S‐CNF composite, demonstrating the uniform distribution of sulfur within the carbon framework. This approach highlights how catalytic hosts can accelerate polysulfide conversion while maintaining structural integrity.

Two‐dimensional materials with tailored phase and chemistry proved to be a powerful platform, exemplified by pre‐lithiated, metallic‐phase 1T‐MoS_2_ nanosheets that synergistically provided high conductivity, strong chemisorption, fast Li^+^ transport, and high catalytic activity, enabling remarkable pouch cell performance [26]. The most sophisticated embodiments of this concept utilize atomic‐scale precision, such as isolated Fe‐Co heteronuclear diatomic sites on hollow carbon spheres, where the Fe and Co sites preferentially catalyze reduction and oxidation steps, respectively, achieving remarkable bifunctional synergy [49]. This evolved further to homonuclear copper dual‐atom catalysts with chlorine bridges, designed to break the activity limit of single‐atom sites and synchronously catalyze sulfur conversion under stringent practical conditions [50]. Further complexity was introduced with transition metal‐bismuth atomic pairs, which were shown to do more than accelerate kinetics; they fundamentally alter the reaction pathway balance to favor direct electrochemical transitions over sluggish chemical processes, offering a key design principle for high‐rate batteries [51]. The accelerated conversion of soluble polysulfides to solid Li_2_S directly addresses the kinetic bottlenecks that cause polarization and incomplete sulfur utilization under lean electrolyte conditions. By reducing the lifetime of soluble intermediates, these promoters also mitigate polysulfide migration and the resulting reaction heterogeneity across the electrode.

#### 2.2.4 Kinetic Promoters for Accelerated Sulfur Redox Kinetics

Beyond merely immobilizing polysulfides, a growing body of research emphasizes the importance of kinetic promoter materials that actively accelerate the sluggish conversion between soluble polysulfides and solid Li_2_S. This catalytic function is critical under practical lean‐electrolyte conditions, where slow kinetics exacerbate polysulfide accumulation and shuttle effects [52]. Various material classes have emerged as effective kinetic promoters. Metal sulfides such as CoS_2_, MoS_2_, and FeS_2_ possess d‐band centers that interact favorably with polysulfide orbitals, lowering activation barriers for conversion reactions. For example, a CoS_2_based sandwiched cathode delivered 1106 mAh g^−1^ after 100 cycles at 0.2 C with a sulfur loading of 4.2 mg cm^−2^. At a high rate of 4 C, the same material retained 823 mAh g^−1^ and showed an ultralow decay of 0.021% per cycle over 1000 cycles [53]. Metal phosphides (e.g., Ni_2_P, Co_2_P, Fe_2_P) trap polysulfides and effectively catalyze Li_2_S decomposition. Metal nitrides combine metallic conductivity with catalytic activity. Vanadium nitride (VN) electrocatalysts enabled lean‐electrolyte operation (E/S ≈ 6 µL mg^−1^) with a high areal capacity of 5.47 mAh cm^−2^ at a sulfur loading of 6.0 mg cm^−2^. The VN@C host delivered 707 mAh g^−1^ at 3.0 C and retained 678 mAh g^−1^ after 400 cycles at 1.0 C [54]. Metal carbides such as Mo_2_C, TiC, and WC also serve as effective kinetic promoters. Mo_2_C nanoclusters on hollow carbon spheres achieved a stable capacity of 904 mAh g^−1^ at 0.5 C under a sulfur loading of 5 mg cm^−2^ and an E/S ratio of 7 µL mg^−1^. The material showed a decay rate of 0.08% per cycle over 400 cycles [55].

Recent advances have focused on atomic‐scale engineering of kinetic promoters. Single‐atom catalysts (SACs) dispersed on conductive hosts maximize active site utilization while minimizing material loading. A Te‐modulated Fe single‐atom catalyst achieved 735 mAh g^−1^ at 5 C and maintained a low decay rate of 0.038% per cycle over 1000 cycles at 1 C [56]. Dual‐atom sites with synergistic effects, such as Fe‐Co heteronuclear pairs, can preferentially catalyze reduction and oxidation steps separately, achieving bifunctional catalysis. A Fe‐Co dual‐atom catalyst exhibited a decay rate as low as 0.018% per cycle over 1000 cycles at 1 C and showed excellent rate performance with 688 mAh g^−1^ at 5 C [49]. Defect engineering introducing sulfur vacancies or heteroatom doping further tunes the electronic structure of promoters. A sulfur‐deficient MoS_2_ catalyst achieved a capacity of 892 mAh g^−1^ at 1 C. Under lean‐electrolyte conditions (E/S = 4 µL mg^−1^) and a high loading of 14.3 mg cm^−2^, the same material delivered an areal capacity of 12.37 mAh cm^−2^ and retained 591.8 mAh g^−1^ after 1200 cycles at 1 C (decay 0.032%/cycle) [57]. These kinetic promoters are most effective when integrated into hierarchical electrode architectures that ensure both rapid ion transport and abundant catalytic sites, highlighting the need for codesign of composition and morphology.

#### 2.2.5 Transport‐Enhancing Architectures

**FIGURE 4:**
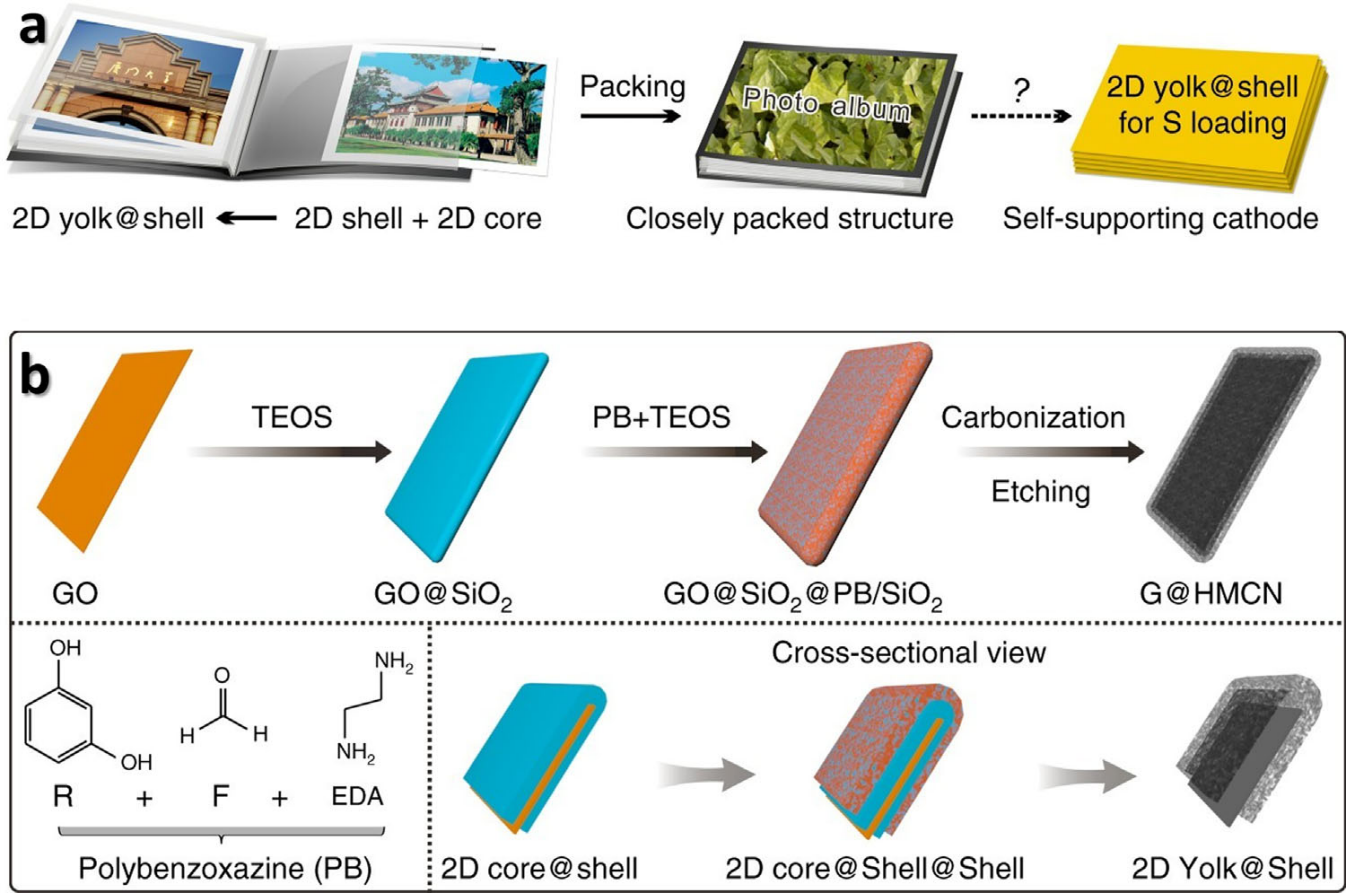
(a) Photo album inspired 2D yolk‐shell nanostructure for the self‐supporting carbon/sulfur cathode. (b) Two‐step coating of GO with SiO_2_ and porous carbon precursor PB/SiO_2_, followed by the carbonization and etching of SiO_2_ to produce G@HMCN. Tetraethylorthosilicate (TEOS) was employed as the SiO_2_ precursor, and resorcinol (R), formaldehyde (F), and ethylenediamine (EDA), were employed as the PB precursors [59]. Adapted with permission from Ref. [59]. Copyright 2017, Springer Nature.

Concurrent with advances in compositional chemistry, significant innovation has focused on the macro‐ and meso‐scale architecture of cathodes to address transport limitations and volumetric strain inherent to high‐loading designs. Early innovative designs focused on creating hierarchical structures, such as a ‘pie‐like’ free‐standing electrode with a multichannel carbon nanofiber core and an amino‐functionalized graphene shell, which demonstrated that areal capacity could be linearly scaled by stacking layers [58]. The concept of eliminating inert components led to binder‐free architectures like sulfur/reduced graphene oxide aerogels [41] and advanced nanostructures such as two‐dimensional carbon yolk‐shell nanosheets, which provided internal void space for volume change and a continuous conductive network, enabling exceptional areal and volumetric capacities [59]. Figure 4a presents a photo album‐inspired schematic of this two‐dimensional yolk‐shell nanostructure, where the 2D yolk and 2D shell create a closely packed structure that forms a self‐supporting cathode for sulfur loading, providing both void space for volume expansion and a continuous conductive pathway. Figure 4b shows the stepwise fabrication process: first, graphene oxide (GO) is coated with SiO_2_ (TEOS as precursor) to form GO@SiO_2_; second, a porous carbon precursor polybenzoxazine (PB), synthesized from resorcinol (R), formaldehyde (F), and ethylenediamine (EDA)) is coated onto the GO@SiO_2_ surface to create GO@SiO_2_@PB/SiO_2_; third, carbonization converts the PB layer into porous carbon; finally, etching of SiO_2_ produces the graphene@hollow mesoporous carbon nanolayer (G@HMCN) structure with a hollow interior. This yolk‐shell architecture exemplifies how nanoscale structural engineering can address both electronic conductivity and volume expansion simultaneously. To overcome ionic transport bottlenecks in thick electrodes, researchers have designed hosts with ordered, three‐dimensionally continuous pore networks. A landmark example is Fe_3_O_4_‐doped carbon cubosomes with a ‘plumber's nightmare’ bicontinuous structure, featuring interpenetrating channels for unimpeded mass transport and a continuous carbon framework for electron conduction [60].

Pushing boundaries further, biomimetic designs have emerged, such as a binder based on hyaluronic acid, whose double‐helix structure creates efficient one‐dimensional ion channels for accelerated Li^+^ migration [61]. A radical topological shift is represented by a modular cathode inspired by neural networks, where individual catalytic “microreactor” modules are interconnected into a fault‐tolerant, fully connected conductive network, effectively decoupling local reaction optimization from long‐range charge transport [62]. Moving beyond static, pre‐assembled structures, transformative paradigms leverage the electrochemical environment to form optimal interfaces in situ. For instance, an amorphous Ni‐B precursor dynamically evolves into a highly active NiSx phase during cycling, creating a self‐optimized catalytic interface [63]. Even more dynamically, photo‐excitable Co/Cu diatomic catalysts can generate transient “dynamic catalytic domains” under light illumination, enabling spatiotemporal modulation of the catalytic microenvironment [64].

The ultimate expression of this design evolution is the holistic integration of conductivity, catalysis, and mass transport in a manufacturable format, explicitly targeting the trifecta of minimizing inert mass, providing multifunctionality, and ensuring lean‐electrolyte compatibility. This is exemplified by the engineering of a lightweight, bifunctional carbon nanofiber host through a steric‐hindrance strategy, which achieved a landmark cell‐level energy density of 502 Wh kg^−1^ in a 2 Ah pouch cell by creating easy‐to‐infiltrate, catalytically active edge sites [65]. Parallel efforts address manufacturability through simplified, integrated synthesis routes, such as the one‐step production of N,S‐codoped porous carbons with precisely tunable sulfur content [66]. Some strategies represent a radical reconceptualization of the cathode itself. One approach eliminates the carbon host and conductive additives entirely by reactivating the current collector, catalytically converting a copper foil into a conductive CuS host in situ, thereby achieving an unprecedented active material content of 95 wt.% [67]. Another frontier moves beyond catalyzing reactions to actively steering the thermodynamic and kinetic landscape of the discharge products themselves, such as using Mo_2_C clusters to promote the formation of two‐dimensional amorphous Li_2_S instead of the insulating crystalline phase, dramatically lowering conversion barriers [68]. Hierarchical pore networks and biomimetic ion channels directly combat concentration polarization by providing low‐tortuosity pathways for Li^+^ transport through thick electrodes. This ensures that active material deep within the cathode remains accessible, promoting uniform sulfur utilization and mitigating the localized overpotentials that accelerate failure.

#### 2.2.6 Multifunctional Binders

**FIGURE 5:**
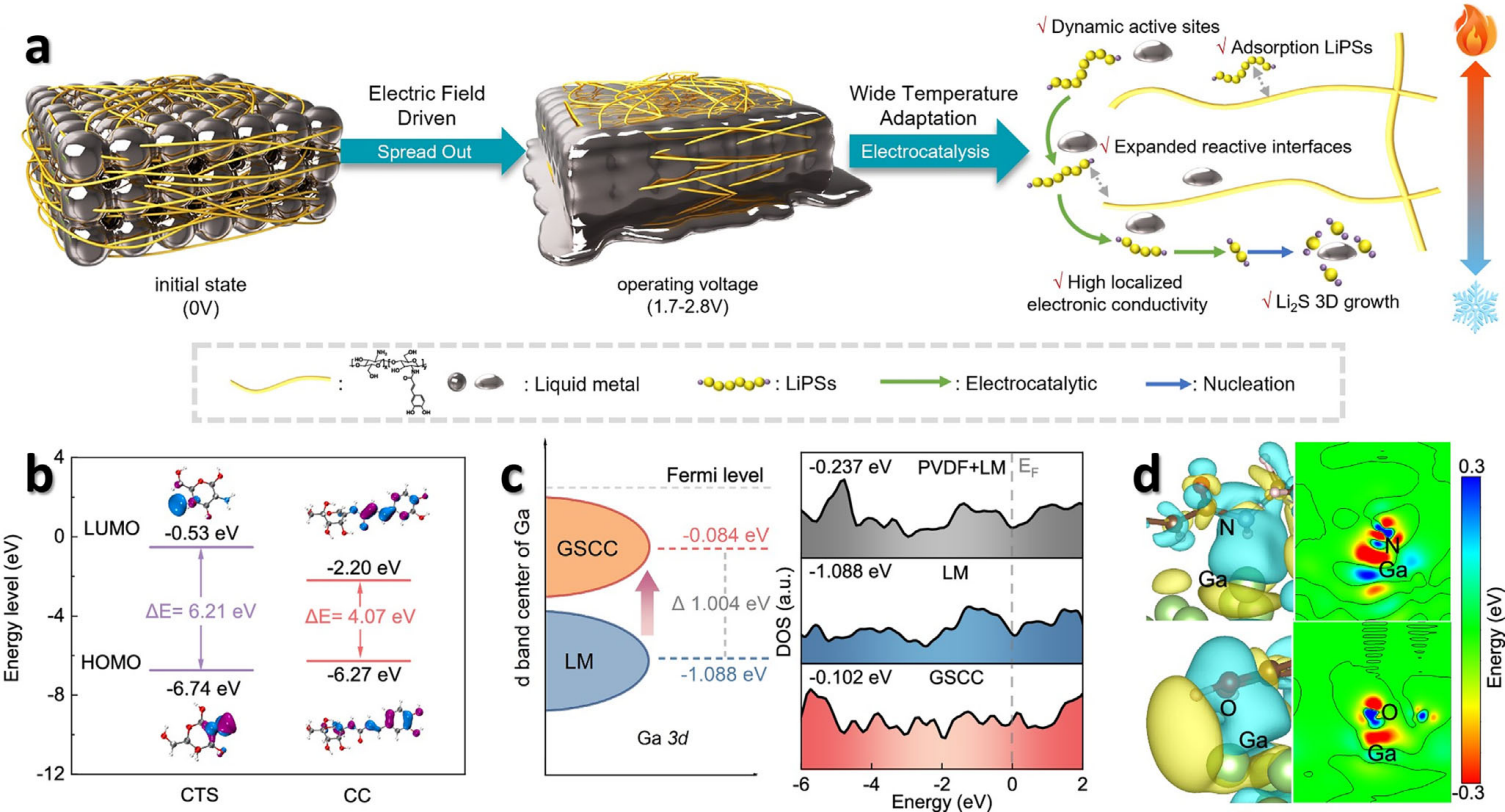
(a) Schematic illustration of GSCC binder's electric field driving characteristics at volt‐free and Li||S battery operating voltages, and the advantages of GSCC‐based Li||S batteries over wide operating temperature ranges. (b) Calculated HOMO and LUMO energy levels of CTS and CC. (c) The *d*‐band center of the *3 d* orbital of Ga atoms in pure LM and GSCC binders and density of states for pure LM, LM + PVDF, and GSCC, *E*_F_ indicates the Fermi energy level (d) Differential charge density modeling and slicing diagram for GSCC. (Blue corresponds to the charge depletion region and red corresponds to the charge accumulation region) [71]. Adapted with permission from Ref. [71]. Copyright 2025, Springer Nature.

Under practical high‐loading conditions, the role of ancillary components becomes as critical as the active host. Binders, in particular, transition from passive adhesives to active, multifunctional elements essential for electrode integrity and electrochemistry. Conventional polyvinylidene fluoride binders fail due to poor polysulfide affinity, lack of ionic conductivity, and inadequate mechanical resilience against large volume changes. Next‐generation binders are therefore engineered for multifunctionality. Sustainable biopolymers like carrageenan offer water‐processability and inherent chemical functionality for polysulfide trapping [69], while hyaluronic acid‐based binders leverage molecular structure to regulate the cathode microenvironment [61, 70]. The pinnacle of this engineering is represented by binders that integrate exceptional functionalities, such as electric‐field‐driven operation with wide‐temperature adaptability, self‐healing capabilities, and intrinsic catalytic activity, effectively transforming the binder from a weak link into a central, adaptive component for all‐climate lithium–sulfur batteries [71]. Figure 5a presents a schematic illustration of the GSCC binder's electric field driving characteristics at volt‐free and Li||S battery operating voltages, highlighting its advantages for Li||S batteries over wide operating temperature ranges. Figure 5b shows the calculated highest occupied molecular orbital (HOMO) and lowest unoccupied molecular orbital (LUMO) energy levels of chitosan (CTS) and chitin (CC), providing insight into the electronic structure and stability of these biopolymer components. Figure 5c displays the d‐band center of the 3d orbital of gallium atoms in pure liquid metal (LM) and GSCC binders, along with the density of states for pure LM, LM+PVDF, and GSCC, where EF indicates the Fermi energy level; the shift in d‐band center correlates with enhanced catalytic activity. Figure 5d presents differential charge density modeling and a slicing diagram for GSCC, where blue regions correspond to charge depletion and red regions correspond to charge accumulation, confirming the favorable charge transfer characteristics at the binder‐electrode interface.

Under practical high‐loading conditions, binders transition from passive adhesives to active components that maintain electrode integrity, provide additional polysulfide anchoring sites, and facilitate ion transport, thereby mitigating mechanical degradation and concentration polarization. This comprehensive evolution in cathode architecture underscores that overcoming the scale‐up imperative requires moving beyond isolated material breakthroughs to the integrated co‐design of composition, nano/micro‐architecture, and ancillary components, all validated under the harsh, lean‐electrolyte paradigm essential for realizing practical high‐energy‐density cells.

#### 2.2.7 Alternative Cathode Chemistries: Metal Sulfides and Organic Sulfides

The preceding discussion has focused on strategies to manage the polysulfide shuttle in conventional sulfur cathodes, where elemental sulfur (S_8_) is hosted within various conductive matrices. An alternative philosophical approach to solving the polysulfide problem is to replace elemental sulfur altogether with materials that inherently avoid soluble polysulfide formation. Two major classes of such materials metal sulfides and organic sulfides have garnered significant attention and represent distinct parallel paths toward practical lithium–sulfur batteries. This section reviews these alternative chemistries and provides a perspective on their respective paths to commercialization. A significant body of work has established various metal sulfides as viable primary cathode materials that operate through conversion reactions without generating soluble polysulfide intermediates. Foundational work on iron sulfide cathodes was reported by Zhu et al., who developed a binder‐free FeS_2_@carbon fiber electrode via electrospinning. By explicitly investigating the Li–FeS_2_ system as a distinct battery chemistry, they revealed its redox mechanisms and achieved remarkable energy densities of approximately 1300 Wh kg^−1^ at the material level, establishing important design principles for metal sulfide electrodes [72].

A conceptually elegant demonstration was reported by Jin et al., who designed a FeS_2_@C nanostructure with a core of iron disulfide and a protective carbon shell. The authors explicitly positioned their work as an alternative to conventional sulfur cathodes, stating that “strict constraint of sulfur to eradicate the polysulfide shuttling is realized in the nanostructure of carbon‐coated metal sulfides instead of pure sulfur cathodes.” This “dual constraint” mechanism combining chemical Fe─S bonding with physical carbon encapsulation enabled stable performance across a wide temperature range from −25°C to 60°C, underscoring practical viability [73]. In parallel to iron sulfides, binary copper sulfide (CuS) has also been demonstrated as a viable cathode active material. Hosseini et al. systematically investigated CuS‐based cathodes in all‐solid‐state configurations. Critically, they highlighted a frequently overlooked advantage of metal sulfide cathodes: their higher density leads to substantially enhanced volumetric capacity. The CuS‐based electrodes achieved volumetric capacities up to 3900 mAh cm^−3^, representing a potential energy density gain of approximately 15% relative to traditional carbon‐sulfur cathodes [74].

Expanding beyond binary systems, a significant advancement was reported by Kızılaslan et al., who synthesized a novel ternary nickel‐cobalt sulfide with NiCo_5_S_8_ stoichiometry for all‐solid‐state batteries. The authors attributed its high performance to three factors: (i) its intrinsically higher electronic conductivity, (ii) its unique morphology, and (iii) its fundamentally different solid‐state conversion mechanism, which they elucidated through in situ Raman spectroscopy and density functional theory (DFT) calculations [75]. Most recently, Pavan et al. provided a sophisticated investigation of FeS_2_, with particular emphasis on the role of electrode microstructure. By systematically comparing processing methods, they demonstrated that homogeneously submicro‐structured composites achieve superior performance, with areal capacities up to 4.28 mAh cm^−2^. Crucially, ex situ analyses revealed that finely structured composites enhance the in situ formation of active material, showing that microstructural engineering is critical for realizing the full potential of metal sulfide‐based cathodes [76].

Beyond pure metal sulfides, some approaches have explored the synergistic combination of metal sulfides with elemental sulfur. The concept was pioneered by Bugga et al., who demonstrated that blending sulfur with TiS_2_ or MoS_2_ forms dense composite cathodes where the metal sulfides function as “redox mediators,” participating actively in the electrochemical reactions rather than serving as passive hosts. They validated this under practically relevant conditions with high sulfur loadings exceeding 12 mg cm^−2^ [77]. Building on this concept, Ulissi et al. investigated C‐FeS_2_‐S composites for all‐solid‐state batteries, demonstrating that metal sulfides and sulfur can function synergistically. By reporting capacities normalized to the combined mass of FeS_2_ and sulfur, they implicitly recognized both components as active participants, achieving areal capacities as high as 3.55 mAh cm^−2^ at practical loadings [78].

Beyond metal sulfides, organic sulfides particularly sulfurized polyacrylonitrile (SPAN) have emerged as a compelling alternative cathode chemistry that fundamentally avoids polysulfide dissolution. Razzaq et al. reported an ultrathin and condensed sulfurized polyacrylonitrile (SPAN) film, highlighting it as “one of the most promising cathode candidates for commercial lithium–sulfur batteries” due to its outstanding capacity reversibility and structural stability. Unlike conventional sulfur cathodes, SPAN undergoes solid‐solid conversion without generating soluble intermediates, enabling stable cycling even in carbonate electrolytes. The resulting cathodes delivered an exceptional initial areal capacity of 8.1 mAh cm^−2^, with a prototype pouch cell achieving 1322 mAh g^−1^ [79]. Comparing these approaches reveals distinct trade‐offs and development timelines. Conventional S_8_/carbon composites offer the highest theoretical energy density but require sophisticated host engineering and lean electrolyte operation to manage polysulfides. Metal sulfides (FeS_2_, CuS, NiCo_5_S_8_) trade some energy density for intrinsic stability and enhanced kinetics, with the added advantage of higher volumetric capacity. SPAN‐based organic sulfides sacrifice some capacity (due to inactive polymer mass) but gain compatibility with existing Li‐ion manufacturing infrastructure and completely eliminate polysulfide chemistry.

In our assessment, SPAN‐based chemistries are closest to near‐term (3–5 year) commercialization, particularly for applications prioritizing cycle life and manufacturing compatibility. Conventional S_8_/carbon composites with advanced catalytic hosts will likely dominate the medium‐term (5–10 year) market for the highest energy density applications (>500 Wh kg^−1^), as they alone can access sulfur's full theoretical capacity. Metal sulfides, while scientifically fascinating and increasingly sophisticated, face fundamental energy density limitations that may relegate them to niche applications or as additives in composite cathodes unless breakthroughs in multi‐electron reactions at higher voltages emerge.

#### 2.2.8 Scalability and Manufacturing Challenges

While the cathode architectures discussed above demonstrate impressive electrochemical performance, their translation to commercial manufacturing presents distinct challenges that are often underappreciated in academic literature. The “scale‐up imperative” demands not only materials that perform under lean electrolyte conditions but also architectures compatible with existing production infrastructure. Slurry‐cast electrodes, which dominate current battery manufacturing, face challenges in achieving uniform dispersion of nanomaterials at scale, with binder migration during drying creating non‐uniform ion transport pathways a problem exacerbated in thick electrodes. Solvent recovery and environmental impact remain significant considerations. Free‐standing electrodes (e.g., carbon foams, electrospun fibers) offer theoretical energy density advantages but are hindered by low volumetric density, poor mechanical robustness in roll‐to‐roll processing, and inconsistent electrolyte wetting. In situ formed electrodes and additive manufacturing approaches demonstrate elegant lab‐scale concepts but face reproducibility, throughput, and quality control challenges when scaled.

Even established materials face hurdles: biomass‐derived carbons contend with batch‐to‐batch consistency variations, while metal‐organic framework (MOF)‐derived materials remain expensive due to costly ligands and multi‐step purification. For the metal sulfide and organic sulfide cathodes discussed in Section 2.2.7, additional considerations arise. Scalable synthesis of phase‐pure metal sulfides requires careful control over temperature, atmosphere, and precursor purity, while SPAN synthesis involves high‐temperature sulfurization processes that must be optimized to ensure uniform sulfur distribution and minimize hazardous byproducts. Both material classes must also demonstrate compatibility with standard electrode processing solvents and binders. The most commercially promising designs will likely balance electrochemical performance with processability favoring architectures that minimize processing steps, utilize industry‐compatible solvents and binders, and demonstrate robust performance across large areas. An additional challenge in thick electrodes is pore blockage, where insoluble discharge products (Li_2_S) or precipitated polysulfides accumulate within the porous network, progressively obstructing ion transport pathways and increasing polarization. This effect is exacerbated under lean electrolyte conditions, where limited solvent volume accelerates pore clogging and leads to premature capacity decay.

### 2.3 The Pouch Cell Reality

The ultimate crucible for any promising cathode material is the multi‐layer pouch cell, a format that introduces complexities entirely absent in coin cells. Coin cells, with their rigid stainless‐steel casing and high, uniform stack pressure, often provide a misleading sense of mechanical stability and interfacial contact. In contrast, pouch cells experience non‐uniform pressure distribution, electrode bending, and gaseous evolution during cycling. Case studies of scaling reveal sobering realities: a nanostructured cathode that demonstrates 1000 stable cycles in a coin cell at low loading may fail rapidly in a pouch cell due to electrolyte depletion a failure mode completely masked in flooded coin cells [80, 81, 82]. Under lean‐electrolyte conditions, any electrolyte consumed in forming unstable interfaces or through parasitic reactions is irreplaceable, leading to sudden cell death. Furthermore, the polysulfide shuttle effect, which may be partially “managed” in a small coin cell, becomes a cascading failure mechanism in a pouch cell. Shuttled polysulfides not only corrode the lithium anode but also migrate throughout the larger cell volume, precipitating in separator pores or inactive areas. This leads to pore clogging, increased polarization, and lithium anode dry‐out. Mechanical degradation is also amplified in pouch cells; the cumulative stress from volume changes in thick, large‐area electrodes can cause progressive delamination from the current collector or cracking within the composite layer, breaking electrical contact. Additionally, current distribution becomes a critical issue inhomogeneities in electrode coating or local impedance variations lead to non‐uniform sulfur utilization and localized over‐discharge/charge, accelerating failure [3, 83].

Successful pouch cell demonstrations, while still rare, consistently share common features. They employ cathodes designed from the outset for high loading and lean E/S, typically utilizing integrated hosts with strong polysulfide affinity and catalytic function. They incorporate optimized, low‐flammability electrolytes such as localized high‐concentration electrolytes (LHCEs) that are used sparingly and offer high stability. Furthermore, they integrate functional separators or interlayers as a failsafe mechanism. A prime example is the macroporous catalytic cathode with double‐binding sites, which, when scaled to a 1‐Ah pouch cell, delivered stable cycling with a specific energy exceeding 300 Wh kg^−1^, validating the transition from coin cell metrics to practical performance [18]. These cases underscore that pouch cell performance is not merely an extension of coin cell data but represents a distinct and more demanding validation stage, revealing failure points rooted in system integration electrolyte balance, interfacial stability, and mechanical engineering that must be addressed through the co‐design strategies discussed in subsequent sections.

**FIGURE 6:**
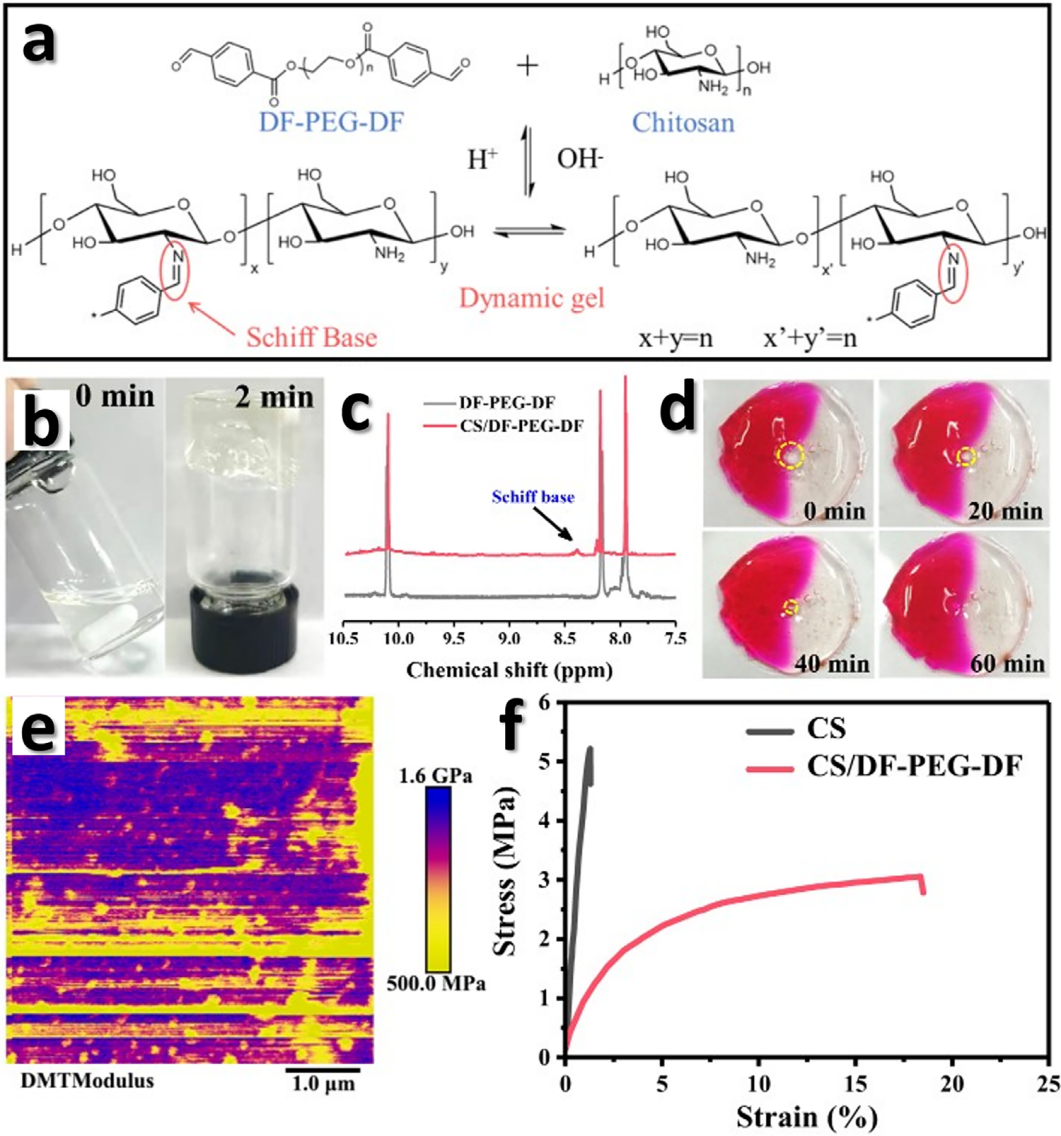
(a) Schematic diagram of the cross‐linking reaction between DF‐PEG‐DF and CS to form gel. (b) Photographs of the CS/DF‐PEG‐DF gel formation process. (c) NMR spectrum of DF‐PEG‐DF and DF‐PEG‐DF with CS in CDCl_3_. (d) The images of self‐healing process of CS/DF‐PEG‐DF at room temperature. (e) Young's modulus mapping of CS/DF‐PEG‐DF film. (f) Stress‐strain curves of CS and CS/DF‐PEG‐DF film [85]. Adapted with permission from Ref. [85]. Copyright 2023, Springer Nature.

The realization of practical high‐energy Li–S batteries is contingent not only on advanced sulfur cathodes but equally on the development of stable lithium metal anodes capable of withstanding repeated cycling. Addressing chronic issues of lithium pulverization and loss of electrical contact, Ye et al. [84] designed an antipulverization, high‐continuity composite anode. Their architecture incorporates solid‐state electrolyte nanoparticles as conformal/sacrificial fillers to guide uniform, dendrite‐free lithium deposition, alongside an embedded copper current collector that ensures mechanical integrity and continuous electron transport. This hybrid anode achieved a high average Coulombic efficiency of ≈99.6% over 300 cycles and, when paired with sulfur cathodes, exhibited high‐capacity retention [84]. Figure 6a presents a schematic diagram of the cross‐linking reaction between difluoro‐poly(ethylene glycol)‐difluoro (DF‐PEG‐DF) and chitosan (CS) to form a dynamic gel via Schiff base formation, where the reversible cross‐linking enables self‐healing behavior. Figure 6b shows photographs of the CS/DF‐PEG‐DF gel formation process at 0 min and 2 min, demonstrating rapid gelation. Figure 6c displays the nuclear magnetic resonance (NMR) spectrum of DF‐PEG‐DF and CS/DF‐PEG‐DF in CDCl_3_, confirming the formation of Schiff base linkages. Figure 6d presents images of the self‐healing process of CS/DF‐PEG‐DF at room temperature at 0 and 20 min, visually demonstrating the material's ability to autonomously repair damage. Figure 6e shows Young's modulus mapping of the CS/DF‐PEG‐DF film via atomic force microscopy, with values ranging from 500.0 MPa to 1.6 GPa, indicating uniform mechanical properties. Figure 6f displays stress‐strain curves of CS and CS/DF‐PEG‐DF films, showing that the cross‐linked gel exhibits enhanced mechanical strength and flexibility compared to pure CS. The design principles developed for Li–S cathodes are proving transferable across different metal‐sulfur chemistries [85]. In a strategy directly parallel to advanced Li–S designs, Zeng et al. [86] engineered a bifunctional host for room‐temperature sodium‐sulfur batteries by coupling nickel single atoms and clusters on a nitrogen‐doped porous carbon monolith. This tandem architecture creates a synergistic catalytic environment that accelerates sodium polysulfide conversion kinetics while simultaneously guiding uniform sodium metal deposition [86].

While the preceding discussion emphasizes the limitations of coin cells in predicting pouch cell performance, it is important to provide a balanced perspective on their continued utility in the research pipeline. Coin cells remain indispensable for rapid material screening, allowing researchers to evaluate novel cathode hosts, electrolyte additives, and binder systems with high throughput and low cost. Their forgiving flooded regime is ideal for isolating intrinsic catalytic kinetics and evaluating initial capacity without the confounding effects of electrolyte starvation. For fundamental mechanistic studies such as probing polysulfide conversion pathways via operando spectroscopy or validating electrocatalyst activity coin cells offer a controlled, well‐understood platform. However, researchers must recognize that promising results in coin cells are necessary but not sufficient for commercial viability. The transition to pouch cell testing should occur once a material system demonstrates stable performance under practically relevant metrics in coin cells (loading >4 mg cm^−2^, E/S <5 µL mg^−1^). Ultimately, coin cells serve as the essential first filter in the development cascade, while pouch cells provide the definitive validation of real‐world performance [87, 88].

### 2.4 From Electrochemistry to Manufacturing: Pouch Cell Assembly and Formation

The translation of Li–S chemistry from a laboratory curiosity to a commercially viable product demands rigorous attention to the engineering processes of cell assembly and the practical realities of the supply chain. This section provides an overview of the key steps involved in fabricating a Li–S pouch cell and discusses the strategic considerations for sourcing its core materials. The assembly of a Li–S pouch cell involves several critical steps that differ from conventional Li‐ion processes. The process begins with the preparation of the sulfur cathode, moving from lab‐scale slurry mixing to industrial‐scale coating techniques. Recent research has successfully demonstrated ultra‐high sulfur loading (up to 12 mg cm^−2^) cathodes using industrially viable, solvent‐free dry‐processing methods that utilize polytetrafluoroethylene (PTFE) binder fibrillation, eliminating toxic solvents and reducing manufacturing costs [89]. After electrode preparation, the stacking and assembly of multilayer pouch cells begins. This typically involves stacking multiple layers of the lithium foil anode, a specialized separator (often ceramic‐coated or functionalized), and the prepared sulfur cathode. The stack is then inserted into an aluminum laminate pouch. The final assembly step is electrolyte filling, which must be performed under strictly controlled conditions (typically a dry room) because of the high reactivity of the lithium metal anode. Unlike Li‐ion cells, Li–S pouch cells require specialized formation protocols during their initial charge‐discharge cycles to establish a stable electrode‐electrolyte interface and activate the cathode. The primary objective of current Li–S battery development is to establish viable manufacturing pathways that can translate laboratory successes into commercially feasible products. During formation, electrochemical studies such as electrochemical impedance spectroscopy (EIS) and cyclic voltammetry (CV) are routinely used to monitor solid‐electrolyte interphase (SEI) formation, detect internal short circuits, and ensure consistent cell quality.

#### 2.4.1 The Supply Chain for Practical Li–S Batteries

The commercial viability of Li–S batteries is heavily dependent on a resilient and cost‐effective supply chain. A strategic advantage of Li–S technology is its ability to decouple performance from the supply chain vulnerabilities and material cost escalations currently plaguing other battery chemistries [90]. The primary materials are abundant and can be sourced ethically. Sulfur is the 10th most abundant element on Earth, and can be sourced as a low‐cost byproduct of petroleum refining, with a market price as low as $0.02/g [5]. Conductive carbon hosts (e.g., carbon nanotubes, graphene) and polymer binders are also required. The high‐energy density relies on a thin lithium metal foil. The supply chain for battery‐grade lithium is rapidly maturing to meet the demands of the broader Li‐ion market, which directly benefits Li–S production. Specialized ether‐based electrolytes (e.g., 1,3‐dioxolane and 1,2‐dimethoxyethane with LiTFSI and LiNO_3_ salts) are required, along with functional separators or interlayers to mitigate the polysulfide shuttle. The backbone of the global Li–S supply chain is beginning to form in specialized industrial zones, particularly in China's Anhui and Guangdong provinces, where proximity to research institutions and material suppliers enables rapid development [91]. Global market research projects the Li–S battery material market to grow from an estimated $0.20 billion in 2025 to $1.7 billion by 2032 [92].

### 2.5 Commercial Cost Comparison and Market Impact

A complete commercial perspective requires a direct comparison between the projected costs of lithium–sulfur (Li–S) batteries and the established costs of lithium‐ion (Li‐ion) batteries, followed by an analysis of how Li–S technology could influence the existing battery market. The fundamental cost advantage of Li–S batteries stems from their raw material composition. Sulfur, the primary cathode material, is the 10th most abundant element on Earth with a raw material cost of just $0.02 per gram, making it dramatically cheaper than cobalt or nickel required in conventional Li‐ion cathodes. Additionally, Li–S cells do not require costly and environmentally challenging rare metals such as cobalt, nickel, and manganese, further reducing material expenses. A comprehensive technoeconomic analysis by Zhong et al. provides the most direct cost comparison between Li–S and Li‐ion batteries under practical conditions. Using a materials‐to‐system analysis based on sub‐Ah level pouch cells with high sulfur loading (6.5 mg cm^−2^) and lean electrolyte (2.5 µL mg^−1^), the authors estimated the cost of Li–S pouch cells to be $60/kWh–$90/kWh, which is visibly lower than the typical commercial Li‐ion battery cost benchmark of $100/kWh [93]. This cost advantage is attributed to three main factors: (i) the use of abundant, low‐cost sulfur and bioderived carbon hosts; (ii) simplified electrode fabrication without mechanical compression; and (iii) reduced electrolyte consumption under lean conditions.

The commercial success of Li–S batteries is unlikely to completely displace Li‐ion technology in the near to medium term. Instead, as noted in a recent perspective, Li–S is expected to complement Li‐ion batteries in the energy storage market [94]. Segments where Li–S may gain market share include weight‐sensitive applications such as aviation and heavy electric vehicles, where the lightweight nature of sulfur offers a distinct advantage [95]. The ultra‐high gravimetric energy density of Li–S with an expected practical specific energy density of approximately 500–600 Wh kg^−1^ makes it ideal for such applications. Furthermore, the use of abundant, low‐cost sulfur aligns with the sustainability goals of grid‐scale storage, where Li–S chemistries are being actively explored for MWh‐scale deployment [96].

Where Li‐ion will likely remain dominant: Li‐ion's mature cycle life (5001 000+ cycles) and established safety track record will be difficult to surpass. Lithium‐iron‐phosphate (LFP) batteries already offer excellent cycle life and safety at competitive costs. Li–S currently lags behind Li‐ion in cycle life (300800 cycles vs. 1 0003 000+ cycles for Li‐ion) [97]. The primary impact of Li–S on the existing Li‐ion market may be supply chain derisking. Li–S technology eliminates dependence on cobalt, nickel, and graphite materials whose supply chains are heavily concentrated in geopolitically sensitive regions. This strategic advantage, combined with lower projected costs, could accelerate investment in Li–S manufacturing capacity alongside continued Li‐ion development.

## 3 The Electrolyte‐Cathode Synergy: An Integrated System

### 3.1 Moving Beyond Isolated Design

The rationale for co‐engineering is rooted in the core failure mechanisms of the Li–S system. The electrolyte is far more than an inert ion‐transport medium; it is the solvent that dictates the solubility, speciation, and mobility of the lithium polysulfide intermediates. The cathode host, in turn, is not a passive container but an active interface that must adsorb, catalyze, and reconvert these species. Designing a cathode for optimal polysulfide chemisorption is futile if the electrolyte solvation structure freely dissolves and shuttles them away [98]. Conversely, formulating an electrolyte that suppresses polysulfide solubility may be ineffective if the cathode host lacks the catalytic sites to facilitate the crucial liquid‐to‐solid conversion, leading to kinetic bottlenecks and passivation. This interdependence is magnified under practical conditions. A lean electrolyte requires a cathode that efficiently retains and reutilizes active material locally, while a thick, dense cathode demands an electrolyte with high lithium‐ion transference number and compatibility with deep pore networks [99]. Therefore, the quest for stability must be recast as a joint optimization problem: the electrolyte must be tailored to complement the surface chemistry and porosity of the cathode, and the cathode must be designed to function optimally within the specific solvation environment created by the electrolyte. This systemic perspective is the cornerstone of modern practical Li–S design [100].

Achieving practical Li–S batteries requires moving beyond the isolated optimization of the cathode or anode to address their highly coupled failure mechanisms, where heterogeneous sulfur conversion directly influences irregular lithium deposition and vice‐versa. To decode this complex interplay, Gao et al. [101] introduced a pioneering quantitative descriptor‐based approach. Inspired by the Butler‐Volmer equation, they identified a binary descriptor (IBD) that combines a mass‐transport index (I_mass_) and a charge‐transfer index (I_charge_) to rationally guide sulfur cathode design. Crucially, they established a direct relationship between this cathode descriptor (IBD) and the morphological evolution of the lithium anode, empirically linking cathode structure to anode stability. Guided by the IBD, they engineered a scalable cathode with interpenetrated channels to ensure homogeneous local current densities. This design, which mitigates reaction heterogeneity on both sides of the cell, enabled an impressive practical energy density of 318 Wh kg^−1^ and 473 Wh L^−1^ in an Ah‐level pouch cell. This work provides a quantitative and mechanistic paradigm for true cathode‐anode co‐engineering, offering a concrete methodology to “unlock the interaction” between electrodes a central tenet of integrated system design [101]. A landmark example is the use of a sulfurized hybrid polymer network with polyphosphazene and carbon [102]. This cathode architecture provides rich sites to re‐bond sulfur species, circumventing the formation of soluble lithium polysulfides and enabling a direct solid‐solid conversion reaction. This fundamental shift, achieved through integrated cathode design, renders the cell inherently resistant to shuttle effects and is a quintessential demonstration of electrolyte‐cathode co‐engineering, yielding stable performance in practical pouch cells [102].

### 3.2 Co‐Engineering Electrolyte and Interface: A Unified Approach to System Stability

To achieve stability under practical conditions, electrolyte design must evolve from a passive, ion‐transport medium to an active, integral component of a co‐engineered system. This section explores advanced strategies from concentrated liquid electrolytes to functional additives and solid‐state systems that are explicitly formulated to stabilize the sulfur cathode and suppress its intrinsic failure mechanisms. A seminal advancement in liquid electrolytes is the development of Localized High‐Concentration Electrolytes (LHCEs). By combining a high concentration of lithium salt (e.g., LiFSI) in ether solvents with a non‐coordinating diluent (e.g., hydrofluoroether), LHCEs create a unique solvation structure.

In this structure, most solvent molecules are tightly coordinated to Li^+^ ions, leaving few free solvents available to dissolve polysulfide intermediates. This dramatically suppresses the shuttle effect at its origin. Furthermore, the anion‐rich primary solvation sheath promotes the formation of a robust, ion‐conductive interphase on both electrodes. The compatibility of this interphase with the cathode surface is critical; while it can form effectively on non‐polar carbons, the aggressive anions in LHCEs may cause corrosive side reactions on polar catalytic hosts (e.g., metal oxides), necessitating tailored surface coatings or electrolyte adjustments. This underscores that LHCE formulation is not a universal solution but must be tuned to the specific cathode chemistry, exemplifying the principle of co‐engineering [99].

Beyond bulk solvent engineering, functional additives serve as precise molecular tools to modify interfacial chemistry and reaction kinetics. The classic additive lithium nitrate (LiNO_3_) operates through a synergistic mechanism, forming a protective passivation layer on the anode while its reduction products can modify the sulfur reduction pathway on the cathode. Newer generation additives are designed with greater specificity. For instance, organosulfur compounds or iodine‐based mediators facilitate the oxidation of insoluble Li_2_S, reducing charge overpotential an effect maximized when the cathode host can adsorb and co‐catalyze the mediator's action. Other additives, like phosphorus‐containing compounds, deliberately react to form a protective, lithium‐ion‐conductive Li_3_PO_4_‐containing cathode electrolyte interphase (CEI), physically blocking polysulfide egress [14, 103]. The efficacy of these molecules is profoundly influenced by the cathode host; a porous carbon may only physisorb them, whereas a polar host with Lewis's acid sites may chemically bind them, altering decomposition pathways and interphase properties. Thus, an additive is not a universal fix but a component of a tailored interface system.

**FIGURE 7:**
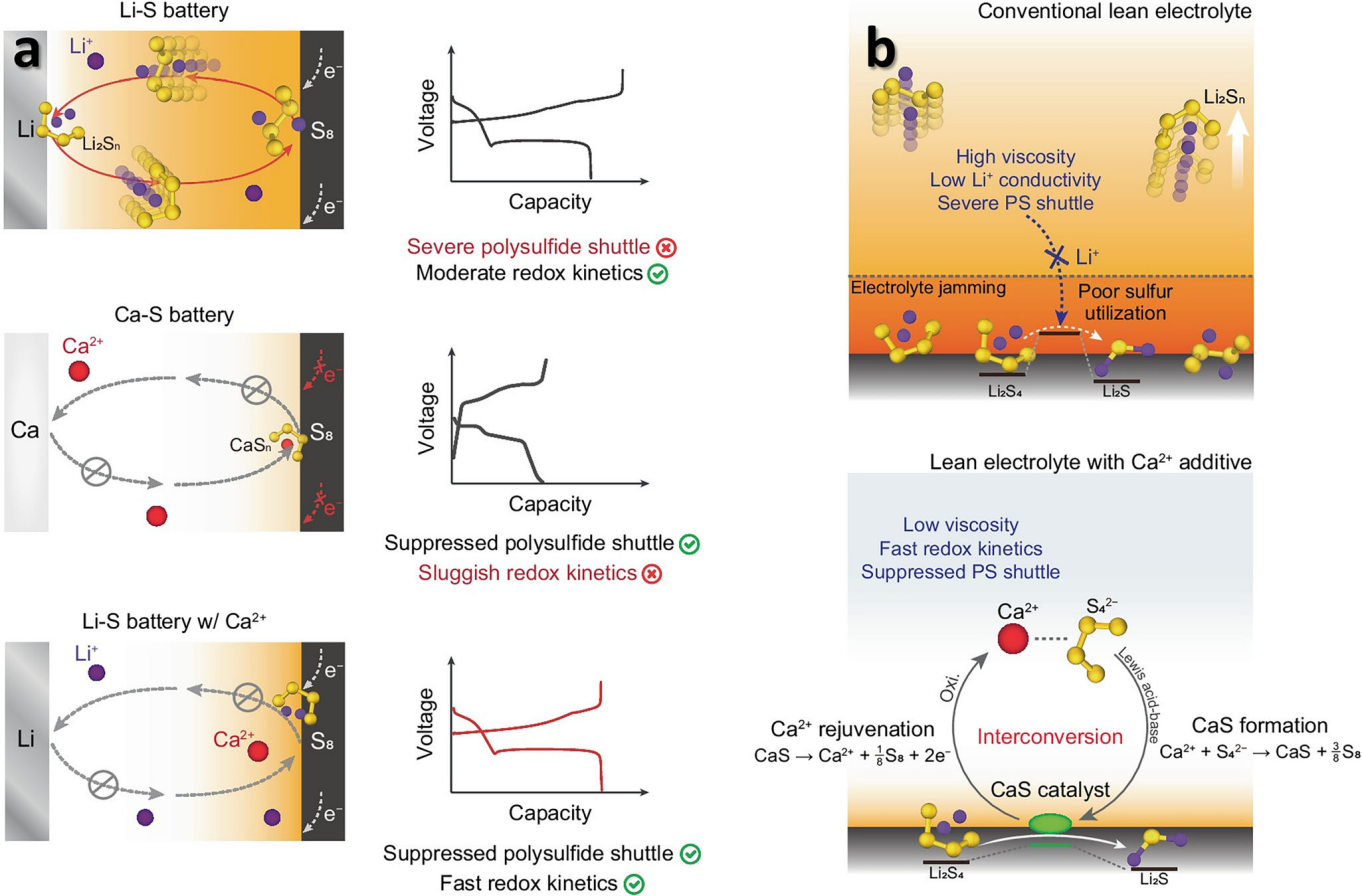
(a) Comparison of LSB, CSB, and LSB with Ca^2+^. LSB with conventional electrolytes shows moderate redox kinetics and significant PS shuttle due to a high PS solubility in the electrolyte. In contrast, CSB maintains a lower PS concentration in their electrolyte phase, thus leading to suppressed PS shuttle but slow redox kinetics. The introduction of Ca^2+^ in the LSB electrolyte effectively mitigates the PS shuttle as well as facilitates the sulfur redox reactions. (b) Design concept of Lewis acidic Ca^2+^ additive for lean electrolyte LSB. Under lean electrolyte conditions, the electrolyte becomes LiPS‐saturated, hindering SRRs and aggravating the PS shuttle. The Ca^2+^ in the electrolyte functions as a PS‐capturing additive, and the in situ‐formed CaS as a catalyst for SRR. The interconversion between Ca^2+^ and CaS makes the dual functions sustainable during operation [105]. Adapted with permission from Ref. [105]. Copyright 2025, Springer Nature.

Building on this, the most advanced additive strategies aim for holistic, system‐wide stabilization by combining molecules with complementary functions. For example, Sun et al. [104] developed a synergistic compound additive integrating LiNO_3_, sodium saccharin, and octaphenyl polyoxyethylene. This cocktail co‐engineers both electrode interfaces, promoting a robust anode SEI while suppressing polysulfide shuttling at the cathode, demonstrating the power of rational multi‐component design [104]. A transformative example of smart molecular design is the use of regenerative, Lewis acidic additives. Cho et al. [105] introduced a Ca^2+^ additive that performs a trifecta of roles: it instantly scavenges polysulfides by precipitating CaS, the resulting CaS acts as a catalyst for sulfur reduction, and the additive is electrochemically regenerated during charging. This sustainable, interconvertible system directly tackles high polysulfide concentrations in lean electrolytes, enabling pouch cells to approach 500 Wh kg^−1^ [105]. Figure 7a provides a comparison of three battery systems: a conventional lithium–sulfur battery (LSB) with moderate redox kinetics and significant polysulfide shuttle due to high polysulfide solubility; a calcium‐sulfur battery (CSB) with lower polysulfide concentration and suppressed shuttle but slow redox kinetics; and an LSB with Ca^2+^ additive, where the additive effectively mitigates the polysulfide shuttle while facilitating sulfur redox reactions. Figure 7b illustrates the design concept of the Lewis acidic Ca^2+^ additive for lean electrolyte LSBs. Under conventional lean electrolyte conditions, the electrolyte becomes lithium polysulfide (LiPS)‐saturated, hindering sulfur reduction reactions (SRRs) and aggravating the polysulfide shuttle. The introduction of Ca^2+^ functions as a polysulfide‐capturing additive (Ca^2+^ + S_4_2^−^ → CaS + S_8_), while the in situ‐formed CaS acts as a catalyst for SRR. The interconversion between Ca^2+^ and CaS (CaS → Ca^2+^ + S_8_ + 2e^−^ during charging) makes these dual functions sustainable during battery operation.

Furthermore, groundbreaking strategies have introduced redox‐active organic molecules as soluble mediators. Li et al. demonstrated that anthraquinone suppresses polysulfide dissolution through a redox‐based chemisorption mechanism, providing strong chemical anchoring akin to polar inorganic hosts [106]. More sophisticated bifunctional molecules, such as 1,3,5‐benzenetrithiol (BTT), are designed to construct stable interfaces on both electrodes simultaneously, reacting in situ to form protective SEI and CEI layers [107]. The most advanced concepts aim to decouple kinetic bottlenecks; Liu et al. introduced a dual redox mediator system using two pseudocapacitive oxides to act as an electron‐ion ‘source’ and ‘drain’ for discharge and charge, respectively, bypassing slow electrochemical steps [108]. An alternative paradigm leverages homogeneous molecular catalysis, as demonstrated by Yang et al. [109] with a soluble binuclear copper complex. This catalyst provides atomically dispersed, fully accessible sites that uniformly guide Li_2_S nucleation throughout the cathode volume, decoupling catalytic function from solid host limitations and enabling stable pouch cell operation [109].

**FIGURE 8:**
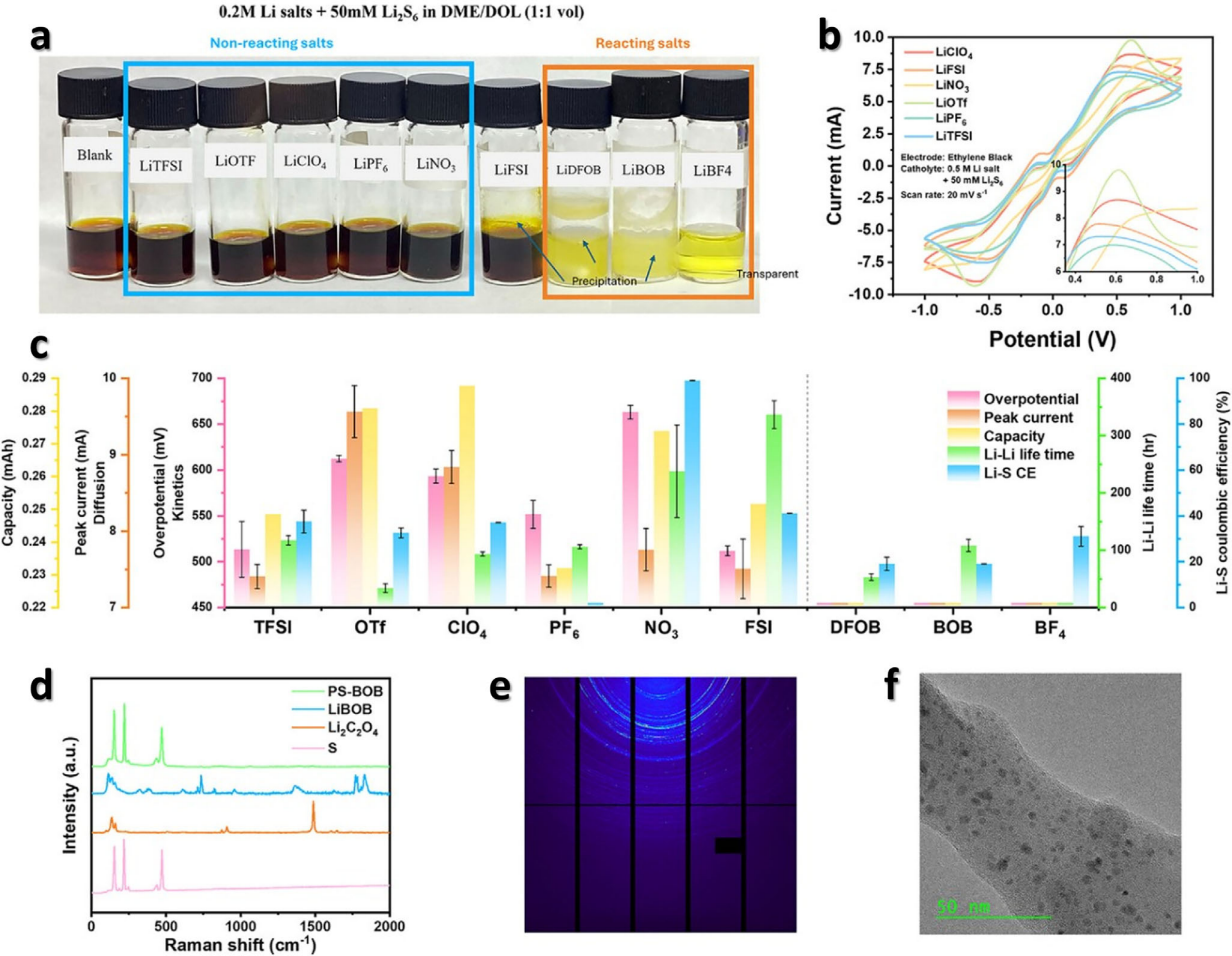
(a) Visualization of the reaction of LiPS and different Li‐salts. (b) CV curves of S–S symmetrical cells with different Li salts. (Inset: Magnified view of CV curves). (c) list of Li‐salt anions and corresponding HOMO/LUMO (top) and electrochemical performance (bottom); (d) Raman spectrum of the reaction product of LiPS and LiBOB (PS‐BOB), LiBOB, Li_2_C_2_O_4_ and S powder. (e) GIXRD of the reaction product of LiPS and LiBOB. (f) TEM image of the reaction product of LIPS and LiBOB [111]. Adapted with permission from Ref. [111]. Copyright 2025, Wiley‐VCH.

While most strategies focus on polysulfide management, some target more fundamental kinetic barriers. Miao et al. [110] identified the desolvation of lithium ions as a critical rate‐limiting step prior to Li_2_S formation. They designed a sulfonate‐group‐rich liquid crystal polymer as a separator modifier; the anionic groups electrostatically attenuate Li^+^‐solvent interactions, lowering the desolvation barrier and accelerating Li_2_S precipitation while simultaneously repelling polysulfide anions [110]. Beyond kinetics, the foundational role of the lithium salt anion itself has been revisited. Fei et al. [111] systematically challenged the preference for LiTFSI, showing that anions traditionally deemed incompatible can be leveraged to form a protective, in situ anion‐derived cathode electrolyte interphase (CEI). This approach delivered outstanding performance under practical conditions and introduced a critical sustainability dimension by questioning the environmental persistence of perfluorinated salts [111]. Figure 8a presents a visualization of the reaction between lithium polysulfides (LiPS) and different lithium salts in DME/DOL (1:1 vol) with 0.2 M Li salts and 50 mM Li_2_S_6_. Non‐reacting salts (Blank, LiTFSI, LiOTF, LiClO_4_, LiPF_6_) show no precipitation, while reacting salts (LiFSI, LiDFOB, LiBOB, LiBF_4_) exhibit visible precipitation, indicating chemical interaction with polysulfides. Figure 8b shows cyclic voltammetry (CV) curves of S–S symmetrical cells with different Li salts at a scan rate of 20 mV s^−1^ using ethylene black electrodes. The inset provides a magnified view, revealing that LiBOB and LiDFOB exhibit higher current responses and lower overpotentials compared to LiTFSI. Figure 8c lists the Li‐salt anions with their corresponding HOMO/LUMO energy levels (top) and electrochemical performance metrics including peak current, overpotential, and Li–S Coulombic efficiency (bottom). The data demonstrate that LiBOB achieves the highest peak current and Coulombic efficiency with the lowest overpotential. Figure 8d displays Raman spectra of the reaction product of LiPS and LiBOB (PS‐BOB) compared to LiBOB, Li_2_C_2_O_4_, and sulfur powder, confirming the formation of oxalate species. Figure 8e presents grazing‐incidence X‐ray diffraction (GIXRD) patterns of the PS‐BOB reaction product, revealing crystalline phases consistent with Li_2_C_2_O_4_. Figure 8f shows a TEM image of the reaction product of LiPS and LiBOB, revealing a uniform nanostructured morphology. Collectively, these results confirm that LiBOB reacts with LiPS to form a protective Li_2_C_2_O_4_‐rich CEI that enhances cycling stability.

The ultimate strategy to eliminate polysulfide dissolution is to adopt solid‐state or quasi‐solid‐state electrolytes. This shift, however, introduces severe interfacial challenges. The rigid solid‐solid contact between a brittle sulfide electrolyte (e.g., Li_6_PS_5_Cl) and a porous, expanding sulfur cathode creates high interfacial resistance and mechanical strain. A more fundamental chemical challenge is the electrochemical oxidation of sulfide electrolytes at sulfur cathode potentials. A landmark study by Yu et al. revealed that this oxidation is initiated by Li^+^ extraction and can be suppressed by using a cathode host that strongly interacts with Li^+^ ions, enabling unprecedented areal capacities in all‐solid‐state Li–S batteries [32].

Successful integration requires meticulous interface engineering and tailoring of the electrolyte's physical properties. Wang et al. designed a low‐density glass‐ceramic solid electrolyte; its low mass allowed a higher volume fraction within the cathode composite at a given weight, creating more robust ion‐conduction pathways and enabling high sulfur utilization [112]. To address mechanical mismatch, self‐healing solid polymer electrolytes with dynamic bonds can autonomously repair cracks and maintain interfacial contact, as demonstrated by Pei et al. [113]. Practical fabrication routes are critical; for example, Kim et al. created an integrated cathode‐electrolyte structure by hot‐pressing, improving contact in a quasi‐solid‐state configuration [114]. The most revolutionary approaches redefine the role of the solid electrolyte from a passive conductor to an active redox mediator. Song et al. designed a lithium thioborophosphate iodide glass where reversible I^−^/I_3_^−^ redox at the surface mediates solid‐solid sulfur conversion, enabling ultrafast charging and exceptional cycle life [115].

Other innovative concepts include the “catalytic solder” strategy, where a material like TiS_2_ chemically fuses the cathode and solid electrolyte into a continuous, catalytically active interface [116], and the design of solid catholytes with intrinsic stabilization mechanisms, such as tungsten‐doped Li_6+x_P_1_‐_x_W_x_S_5_I, where in situ formed WS_2_ promotes reversible interphase chemistry [117]. A radical alternative to ethers is the use of carbonate‐based electrolytes, standard in Li‐ion batteries. Long considered incompatible, operando studies revealed that in carbonates, sulfur undergoes a direct solid‐phase conversion to Li_2_S, bypassing soluble polysulfide formation altogether. This insight has enabled the design of sulfur cathodes that cycle stably in carbonate electrolytes, opening a distinct, shuttle‐free pathway [118].

**FIGURE 9:**
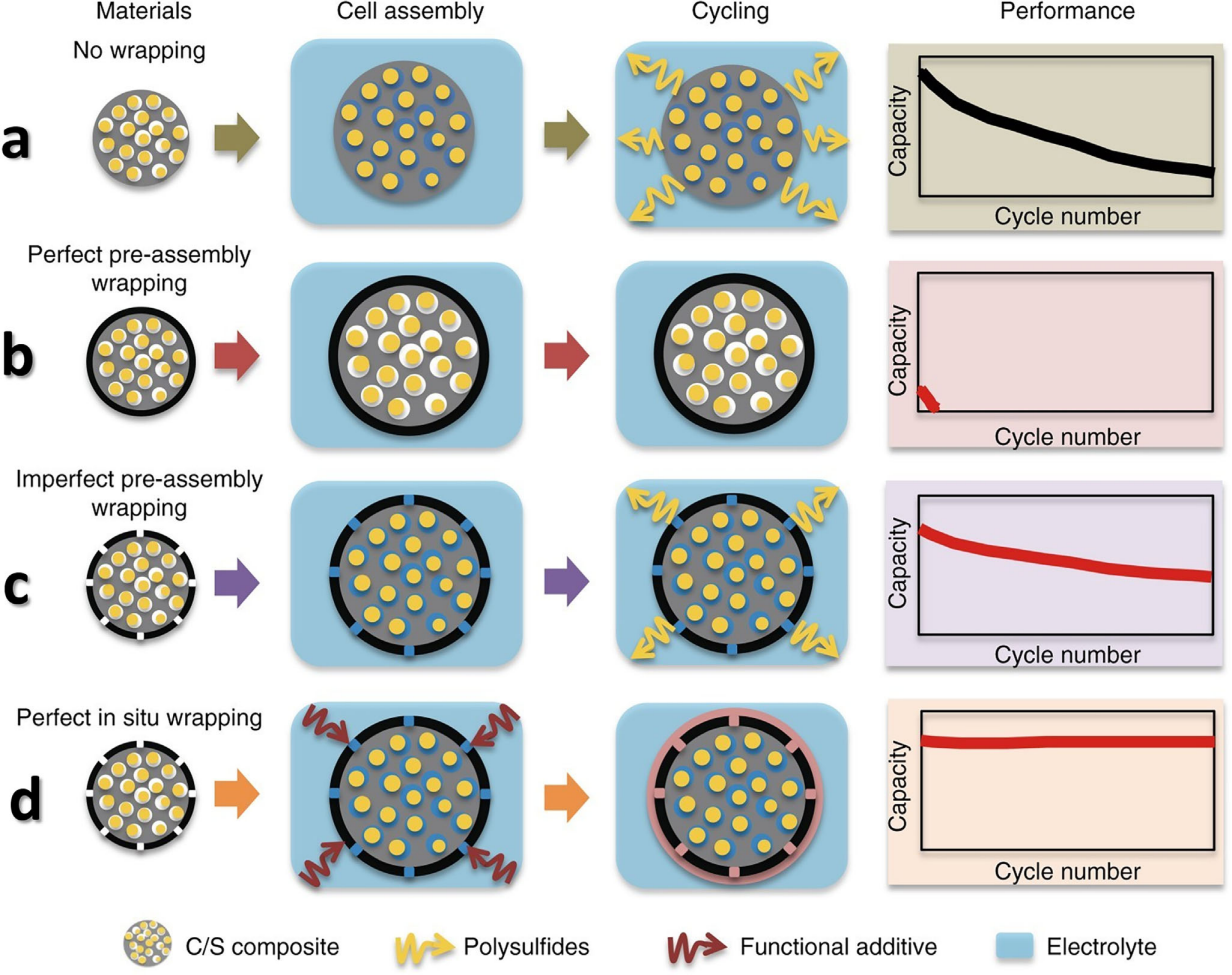
(a) The no wrapping case, which exhibits severe capacity decay during cycling. (b) Perfect wrapping of the C/S materials prior to battery assembly, which exhibits poor overall performance due to the lack of electrolyte in the active material. (c) Imperfect wrapping of the cathode material prior to battery assembly, which exhibits improved cycle stability compared with the no wrapping case. (d) Perfect post‐assembly in situ wrapping of the cathode material, which exhibits ideal cycle stability using a blocking polysulfide shuttle while allowing for electrolyte infiltration in the active material [121]. Adapted with permission from Ref. [121]. Copyright 2017, Springer Nature.

Recognizing that the critical reactions occur at interfaces has led to the strategic development of engineered barriers and functional layers that are best understood as extrinsic, multifunctional extensions of the cathode system. These components including cathode coatings, functional separators, and interlayers do not replace the need for an optimized host or electrolyte but provide a critical line of defense and regulation, dynamically interacting with both to manage species transport, define reaction zones, and prolong cell life. The most direct form of interface engineering involves applying thin, conformal coatings directly onto the sulfur composite electrode. These layers composed of polymers (e.g., PAN, PEDOT), metal oxides (e.g., Al_2_O_3_ via ALD), or carbon serve multiple roles: they act as a physical sieve, selectively permitting Li^+^ transport while blocking larger polysulfide anions; they provide secondary chemical adsorption sites; and they stabilize the electrode surface against electrolyte decomposition. Early work established the viability of this approach, such as encapsulating sulfur nanoparticles in a conductive PEDOT shell to improve electronic contact and act as a polysulfide diffusion barrier [119]. Similarly, combining conductive polymers with carbon nanostructures for example, wrapping a sulfur/single‐walled carbon nanotube composite with a polyaniline web demonstrated how such coatings can enhance cycle life by providing both a barrier and conductivity [120]. A critical design trade‐off exists between creating an impermeable barrier and maintaining electrolyte infiltration and ionic pathways. Advanced strategies therefore focus on creating intelligent, in situ formed layers that balance these needs. For instance, Hu et al. [121] developed an in situ wrapping technique that constructs an intentionally imperfect, compact layer on carbon/sulfur particles. This design strategy suppresses polysulfide egress while allowing beneficial internal diffusion and reaction, resolving the core performance trade‐off and enabling exceptional long‐term stability [121]. Figure 9a illustrates the “no wrapping” case, where bare C/S composite particles are directly assembled into a cell. Without any protective layer, polysulfides freely shuttle between electrodes, leading to severe capacity decay during cycling. Figure 9b shows the “perfect pre‐assembly wrapping” case, where C/S particles are fully encapsulated with a conformal coating prior to cell assembly. While this approach effectively blocks polysulfide egress, it also prevents electrolyte infiltration into the active material, resulting in poor overall performance due to ionic transport starvation. Figure 9c presents the “imperfect pre‐assembly wrapping” case, where C/S particles are intentionally coated with an incomplete or porous layer before assembly. This design allows partial electrolyte access while providing some polysulfide blocking capability, leading to improved cycle stability compared to the no‐wrapping case, though optimal performance is not yet achieved. Figure 9d depicts the “perfect in situ wrapping” case, where the protective layer forms dynamically after cell assembly through reaction with electrolyte components or functional additives. This post‐assembly wrapping achieves ideal cycle stability by effectively blocking the polysulfide shuttle while simultaneously allowing complete electrolyte infiltration into the active material, thereby resolving the permeability‐barrier trade‐off.

**FIGURE 10:**
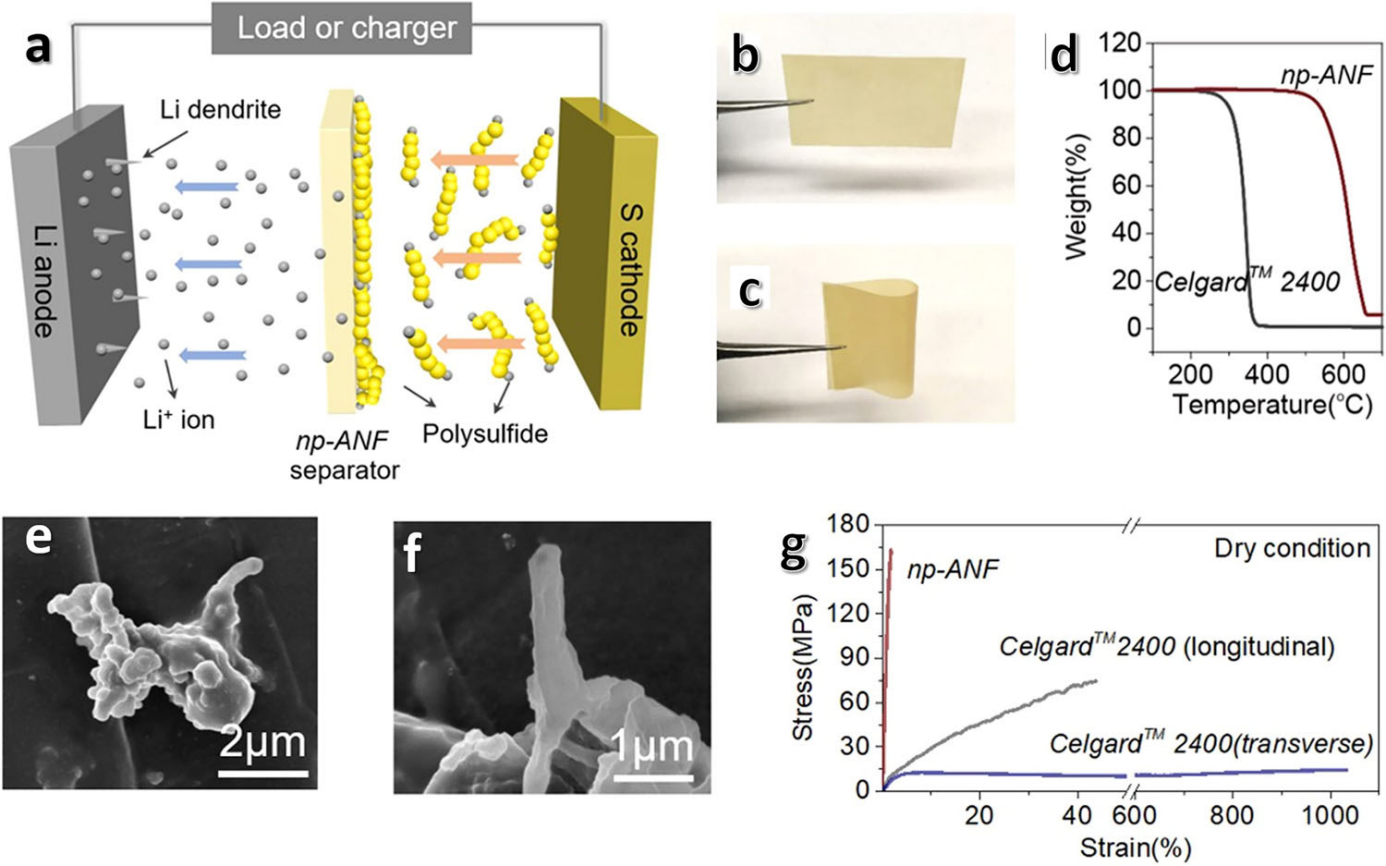
(a) Schematic configuration of a Li–S cell with a *np‐ANF* membrane between the sulfur cathode and the lithium anode. (b,c) Photographs of an *np‐ANF* membrane. (d) TGA curves for *np‐ANF* membrane and *Celgard* 2400. (e,f) SEM images of the tip of lithium dendrite. (g) Stress‐strain curves for *np‐ANF* and *Celgard* 2400 [124]. Adapted with permission from Ref. [124]. Copyright 2022, Springer Nature.

More prevalently, functional separators and interlayers are employed as a pragmatic, scalable strategy for system‐level polysulfide management. Placed between the cathode and the separator, a lightweight interlayer of microporous carbon, graphene, or polar composites acts as a polysulfide “trap” and a secondary current collector, recapturing shuttling species and facilitating their reconversion. However, in lean‐electrolyte cells, an interlayer's pore volume can become an undesirable electrolyte reservoir, reducing the effective E/S ratio in the main cathode. Modern designs therefore prioritize ultrathin, dense, and highly catalytic interlayers that maximize adsorption sites with minimal volume and electrolyte uptake. The functionality of these layers has evolved significantly. Early innovations involved bifunctional designs like a BN‐carbon trilayer separator, where a carbon layer facing the cathode traps polysulfides and a boron nitride layer facing the anode provides a protective barrier against polysulfide corrosion while promoting uniform lithium plating [122]. The most advanced designs aim for precise ionic regulation. For example, a separator coated with negatively charged Ti_0_._87_O_2_ nanosheets featuring atomic vacancies electrostatically repels polysulfide anions to suppress shuttle, while the vacancies act as ion‐selective nanopores to homogenize Li^+^ flux, thereby stabilizing both electrodes [123]. Another transformative approach utilizes crystalline porous materials as molecular sieves. Bioinspired designs offer a route to multifunctionality. Wang et al. engineered a cartilage‐like membrane from aramid nanofibers that creates an ion‐selective, negatively charged barrier to block shuttle while its mechanical robustness suppresses lithium dendrite growth, addressing both major failure modes in a single component [124]. Figure 10a presents a schematic configuration of a Li–S cell incorporating a nanoporous aramid nanofiber (np‐ANF) membrane placed between the sulfur cathode and the lithium anode. The membrane functions as an ion‐selective barrier, blocking polysulfide shuttling while allowing Li^+^ transport. Figure 10b,c shows photographs of an np‐ANF membrane, demonstrating its free‐standing nature and mechanical flexibility. Figure 10d displays thermogravimetric analysis (TGA) curves for the np‐ANF membrane compared to Celgard 2400, revealing that the np‐ANF membrane exhibits enhanced thermal stability with higher decomposition temperature. Figure 10e,f presents SEM images of the tip of a lithium dendrite, showing the sharp, needle‐like morphology that can penetrate conventional separators and cause internal short circuits. Figure 10g shows stress‐strain curves for np‐ANF and Celgard 2400 under dry conditions. The np‐ANF membrane exhibits significantly higher mechanical strength and elongation at break compared to Celgard 2400 in both longitudinal and transverse directions, providing superior resistance to dendrite penetration.

To mitigate the penalty of adding inactive mass, innovative “active interlayers” have been developed. Lee et al. designed an interlayer from sulfur‐impregnated polar mesoporous silica, where the silica framework chemically traps polysulfides and the embedded sulfur acts as an additional active material reservoir, contributing directly to capacity [125]. The development of advanced fabrication techniques has enabled more precise and scalable interface engineering. Initiated Chemical Vapor Deposition (iCVD) allows for the application of conformal, ultra‐thin polymer coatings directly onto sulfur cathodes. As demonstrated by Lee et al., a super‐stretchable copolymer coating applied via iCVD accommodates large volume changes without cracking and reduces polysulfide shuttling, showcasing how scalable coating technologies can create mechanically adaptive interfaces [126]. Translating these concepts into commercially relevant formats requires system‐level solutions that perform under extreme conditions. Huang et al. [127] developed a multifunctional separator integrating a Ti_3_C_2_T_x_ MXene@CuCo_2_O_4_ composite on a standard polyethylene substrate. This design provides strong adsorption and catalytic conversion of polysulfides while using the conductive MXene framework to regulate Li^+^ flux for uniform lithium deposition. Deployed in a large‐format 10 Ah pouch cell under stringent conditions (10 mg cm^−2^ sulfur, E/S = 2.6 µL mg^−1^), the cell achieved 417 Wh kg^−1^, demonstrating that intelligent interface engineering can realize high energy density in commercially significant formats [127]. Addressing the formidable challenge of wide‐temperature operation, Li et al. [128] engineered a multifunctional interface through molecularly designed polymer brushes grafted onto graphene. This interface synergizes three actions: anthraquinone groups act as a redox mediator, the dense brush structure provides a shuttling barrier at high temperatures, and the same structure homogenizes Li^+^ flux at low temperatures. This triple‐function synergy enabled stable operation from −40°C to 70°C in an Ah‐level pouch cell, illustrating the power of macromolecular engineering to create adaptive interfaces for all‐climate batteries [128]. In summary, interface engineering has evolved from simple physical barriers to sophisticated, multifunctional components that are integral to the co‐engineered Li–S system. Through strategic design at the molecular, nano‐, and macro‐scale, these interfaces actively manage the complex interplay of ionic transport, chemical conversion, and mechanical stress, providing essential pathways to stabilize performance under the demanding conditions of practical high‐energy‐density cells.

The critical nature of this performance chasm is exemplified by materials engineered specifically to bridge it. A case in point is the work of Qian et al. [55], who designed a hierarchical host of Mo_2_C nanoclusters on carbon hollow spheres (Mo_2_C/CHS) explicitly to address the kinetic and transport limitations of practical cells. While this catalytic host delivers impressive performance, its reported metrics are highly instructive: it achieves a stable discharge capacity of ∼904 mAh g^−1^ at 0.5 C only under conditions of a sulfur loading of 5 mg cm^−2^ and an E/S ratio of 7 µL mg^−1^. This study is a prime example of research targeting the “harsh paradigm,” yet it also underscores the remaining gap, as the E/S ratio used, while improved, is still above the stringent <3 µL mg^−1^ required for the highest cell‐level energy densities. This highlights the continuous gradient of practicality and demonstrates that even advanced materials must be evaluated against the most rigorous benchmarks to prove true viability for commercialization [55]. The pinnacle of separator engineering lies in creating a single component that actively and simultaneously addresses failures on both sides of the cell. Demonstrating this holistic approach, Kong et al. [129] designed a Co/MoN composite as a separator modifier (Co/MoN@PP) to invigorate kinetics bilaterally. On the cathode side, the material provides strong adsorption and catalytic conversion of polysulfides, effectively suppressing shuttle and enhancing redox reversibility. Concurrently, on the anode side, it functions as a Li^+^ flux regulator, promoting uniform ion distribution to enable dendrite‐free lithium plating/stripping for over 800 h. This synergistic modulation resulted in exceptional full‐cell performance, including an areal capacity of 4.62 mAh cm^−2^ under a high sulfur loading of 4.92 mg cm^−2^. This work exemplifies how a rationally designed interfacial component can serve as a central, active hub for co‐regulating the interdependent cathode and anode electrochemistry, moving beyond simple barrier functions toward integrated system control [129].

From a commercial perspective, the translation of these advanced separators and interlayers from laboratory demonstrations to practical applications requires careful consideration of cost, scalability, and manufacturability. Materials that involve expensive precursors, multi‐step synthesis, or complex coating processes may face barriers to adoption in large‐format cell production. Ideally, commercially viable designs should utilize industry‐compatible substrates (e.g., polyolefin separators), employ scalable coating techniques (e.g., slot‐die coating, gravure printing), and minimize additional processing steps. Furthermore, the mechanical robustness and thermal stability of these layers under roll‐to‐roll manufacturing and cell assembly conditions must be validated to ensure consistent performance across large electrode areas.

#### 3.2.1 Cathode‐Anode Crosstalk: The Missing Link in Integrated Design

Perhaps the most underappreciated aspect of Li–S system integration is the bidirectional communication or crosstalk between the sulfur cathode and lithium metal anode. Species generated at one electrode profoundly influence the behavior of the other, creating feedback loops that can accelerate failure or, if properly managed, enable synergistic stabilization. Recognizing and engineering this crosstalk is essential for true “integrated design.” The most obvious form of crosstalk is the polysulfide shuttle itself: soluble polysulfides generated at the cathode migrate to the anode, where they corrode the lithium metal, forming a thick, resistive solid‐electrolyte interphase (SEI) and consuming both lithium and electrolyte. This not only degrades the anode but also depletes the cathode of active material. Conversely, species generated at the anode such as decomposed electrolyte fragments or lithium‐containing complexes can migrate to the cathode and modify its surface chemistry, potentially altering catalytic activity or passivating active sites. Recent work has revealed more subtle forms of crosstalk. Transition metal dissolution from catalytic hosts (e.g., Mn, Co, Fe) can occur under certain electrochemical conditions; these dissolved metal ions migrate to the anode, where they are reduced and incorporated into the SEI, altering its ionic conductivity and mechanical properties. Conversely, anode‐derived species such as lithium hydride or lithium nitride (from nitrogen‐containing electrolyte additives) can migrate to the cathode and participate in sulfur conversion reactions, potentially enhancing kinetics.

Perhaps most critically, reaction heterogeneity at the cathode directly translates to inhomogeneous lithium deposition at the anode. As demonstrated by Gao et al. [101], non‐uniform sulfur conversion creates localized variations in current density and lithium‐ion flux, which in turn induce irregular lithium plating and stripping. This coupling means that cathode designs promoting uniform reaction distribution (e.g., interpenetrating conductive networks, graded porosity) simultaneously stabilize the anode a powerful example of crosstalk‐mediated co‐engineering. Recognizing this interdependence, the most advanced system‐level designs now explicitly target bidirectional regulation. Functional separators modified with materials like Co/MoN composites simultaneously catalyze polysulfide conversion at the cathode‐facing side while homogenizing Li^+^ flux at the anode‐facing side, as demonstrated by Kong et al. [129]. Electrolyte additives such as LiNO_3_ form protective layers on both electrodes through distinct decomposition pathways. The emerging paradigm, therefore, is not simply to optimize cathode and anode independently, but to engineer the entire system such that reactions at one electrode actively support stability at the other a true symbiotic electrochemical system. This bidirectional regulation has translated to tangible performance improvements: Co/MoN‐modified separators enabled an areal capacity of 4.62 mAh cm^−2^ under high sulfur loading of 4.92 mg cm^−2^, with dendrite‐free lithium plating for over 800 h [129].

### 3.3 Electrolyte Safety and Thermal Runaway in Pouch Cells

While the previous sections focused on electrolyte design for suppressing the polysulfide shuttle and enhancing electrochemical performance, a critical but often overlooked aspect is the influence of liquid electrolytes on the thermal safety of Li–S pouch cells. The commercial viability of Li–S batteries demand not only high energy density and long cycle life but also robust safety performance under both normal and abusive conditions. In Li–S pouch cells, the liquid electrolyte plays a decisive role in determining thermal runaway behavior through three interconnected mechanisms.

Conventional Li–S electrolytes are based on ether solvents such as 1,3dioxolane (DOL) and 1,2dimethoxyethane (DME), which possess low flash points and boiling points. Consequently, these electrolytes are inherently flammable and prone to decomposition at elevated temperatures. Moreover, the dissolved lithium polysulfides inevitable intermediates in Li–S chemistry exhibit temperature‐dependent reactivity. At elevated temperatures, higher‐order polysulfides (Li_2_S_x_, x ≥ 6) become more reactive and can trigger strong exothermic reactions with the lithium metal anode. A landmark study by Jiang et al. [130] revealed that the reactions between dissolved higher‐order polysulfides and lithium metal are the primary origin of thermal runaway in 1.0 Ah cycled Li–S pouch cells. Crucially, this study demonstrated that a 16cycle pouch cell remained safe when heated from 30 to 300°C without additional electrolyte, but severe thermal runaway occurred at 147.9°C when extra electrolyte was added. This counterintuitive finding highlights the dual role of the electrolyte: while necessary for ion transport, excess electrolyte can exacerbate thermal hazards by facilitating polysulfide mobility and providing additional fuel for exothermic reactions.

Under lean‐electrolyte conditions (E/S ≤ 3 µL mg^−1^), a remarkable safety feature emerges. Systematic thermal safety assessments have revealed that Li–S pouch cells with lean electrolyte volumes exhibit significantly suppressed thermal runaway. The mechanism behind this behavior is multifaceted. First, the high viscosity of a lean electrolyte under thermal stress leads to poor solvent transport at the electrode‐electrolyte interface, which paradoxically enhances the thermal stability of battery components at high temperatures. Second, the limited electrolyte volume becomes quickly consumed by parasitic reactions at both electrodes during initial cycling, forming stable interphases that act as physical barriers against further exothermic reactions. Third, as the electrolyte depletes, the concentration of dissolved higher‐order polysulfides (Li_2_S_x_ ≥ 6) the most dangerous species for triggering runaway remains low. In contrast, pouch cells with excess electrolyte maintain high concentrations of these reactive polysulfides, providing the necessary “fuel” for catastrophic thermal runaway. The specific sulfur species present in the cycled electrolyte dictate the thermal behavior of Li–S pouch cells. Jiang et al. [130] systematically analyzed the electrolyte composition of cycled cells and found that higher‐order polysulfides (Li_2_S_x_ ≥ 6) are the key culprits driving thermal runaway. Strong exothermic reactions between cycled lithium metal and dissolved Li_2_S_6_/Li_2_S_8_ were observed at approximately 153.0°C, directly initiating runaway. In contrast, when the sulfur species in the electrolyte are predominantly lower‐order polysulfides (Li_2_S_x_ ≤ 4), thermal runaway does not occur even with the addition of extra electrolyte. This discovery underscores that suppressing the polysulfide shuttle is not only critical for electrochemical performance but also a fundamental safety requirement.

#### 3.3.1 Strategies for Enhancing Electrolyte Safety

Several promising electrolyte engineering strategies have emerged to mitigate thermal runaway risks. LHCEs have demonstrated enhanced thermal stability compared to conventional dilute electrolytes. By reducing the amount of free solvent molecules available for decomposition, LHCEs suppress side reactions and improve the thermal durability of the electrolyte. However, recent studies caution that LHCEs can still fail at temperatures above 80°C due to uncontrolled reductive decomposition of Lian‐ion aggregates on the lithium anode side [131]. This limitation necessitates further optimization of solvation structures for high‐temperature applications. Incorporating flame‐retardant functional groups into the electrolyte or binder system can significantly improve safety. For instance, multifunctional binders with intrinsic flame‐retardant properties have been shown to effectively suppress combustion and enhance the thermal safety of Li–S batteries [132]. A particularly innovative approach involves designing a “smart” thermoresponsive SEI using electrolyte additives. Jiang et al. demonstrated that a lithium iodide additive in the electrolyte not only enhances electrochemical performance under normal conditions but also triggers the self‐assembly of a dense antiperovskite layer on the lithium surface at elevated temperatures. This thermally stable inorganic layer greatly inhibits exothermic reactions, raising the thermal‐runaway onset temperature of pouch cells from 116.0°C to 162.3°C. Such strategies provide novel pathways to simultaneously enhance safety and lifespan [133].

In summary, the liquid electrolyte in Li–S pouch cells is a double‐edged sword: it is essential for ionic transport but also represents the primary source of thermal risk. Lean‐electrolyte conditions, while challenging for performance, offer inherent safety advantages by minimizing the “fuel” available for exothermic reactions. The future of safe Li–S batteries lies in the rational co‐engineering of electrolyte solvation structures, the suppression of higher‐order polysulfide accumulation, and the incorporation of smart thermal‐responsive additives that can actively protect the cell under abuse conditions.

## 4 Data‐Driven Design and Performance Benchmarking

### 4.1 The Multi‐Parameter Optimization Problem

The performance of a Li–S cell emerges from a non‐linear and often competing set of parameters. Central to this is the triad of sulfur loading (L), electrolyte‐to‐sulfur ratio (E/S), and electrode porosity (Φ), which collectively govern the system's electrochemistry. Increasing *L* boosts areal capacity but elevates ionic/electronic resistance and mechanical stress. Decreasing E/S is essential for energy density yet starves the reaction of ionic carriers, exacerbates concentration polarization, and can lead to rapid failure if not perfectly managed. Optimizing *Φ* involves a delicate balance: sufficient pore volume is needed to accommodate sulfur and its ∼80% expansion, but excessive porosity reduces volumetric energy density, increases electrode tortuosity, and traps excess electrolyte, effectively raising the local E/S [134].

These primary variables are further modulated by secondary ones: the electronic conductivity of the host matrix, the catalytic activity and affinity of host surfaces for polysulfides, the ionic conductivity and solvation structure of the electrolyte, and the mechanical properties of the binder. The relationship between these inputs and key outputs initial areal capacity, capacity retention, Coulombic efficiency, and energy density is highly coupled [135]. A change in one parameter, such as introducing a more catalytic but less conductive host, can shift the optimal values for E/S and porosity. Visualizing this landscape requires moving beyond two‐dimensional plots to multi‐axis charts or parallel coordinate plots, where “sweet spots” appear as narrow regions where multiple performance metrics simultaneously meet practical targets [136].

Recognizing this complexity is the first step toward a systematic rather than serendipitous search for solutions. The critical yet often overlooked role of porosity was definitively established by Kang et al. [137]. They demonstrated experimentally and through modeling that decreasing cathode porosity from 70% to 40% has a profound impact on polarization, capacity, and cycle life. Their work revealed that sulfur utilization is limited by polysulfide solubility at high porosity, while conversion to Li_2_S is limited by electronic surface area at low porosity, leading to a predicted optimal porosity that maximizes volumetric energy density. This study crystallized porosity as a key variable in the multi‐parameter optimization problem [137].

### 4.2 The Role of Advanced Characterization and Modeling

To move beyond correlation toward causation, and to generate the high‐fidelity data needed for predictive models, advanced characterization and multi‐physics modeling are indispensable. *Operando* and in situ techniques provide dynamic, mechanistic insights that static, post‐mortem analysis cannot, while computational modeling transforms qualitative design heuristics into quantitative design rules. Together, these tools form a feedback loop that elucidates fundamental mechanisms, validates material performance, and guides rational engineering. *Operando* techniques are crucial for tracking dynamic processes in real time. X‐ray diffraction (XRD) and X‐ray absorption spectroscopy (XAS) monitor the crystallographic phase evolution of sulfur species within a working cathode, identifying conditions that lead to passivating crystalline Li_2_S formation. Operando Raman spectroscopy and UV–vis quantify the concentration and speciation of polysulfides in the electrolyte, directly measuring the efficacy of shuttle suppression strategies. Advanced scattering techniques, such as operando small‐angle/wide‐angle X‐ray scattering (SAXS/WAXS) and small‐angle neutron scattering (SANS), provide insights into nucleation, growth, and dissolution of solid discharge products from the atomic to sub‐micron scale. A definitive study by Prehal et al. used these methods to reveal that solid short‐chain polysulfides coexist with Li_2_S and that Li_2_S forms via solid‐state reduction of precipitated Li_2_S_2_, conclusively demonstrating that mass transport, not electron transport through a passivating layer, is the primary rate‐limiting factor a finding that fundamentally guides electrode and electrolyte design [138].

**FIGURE 11:**
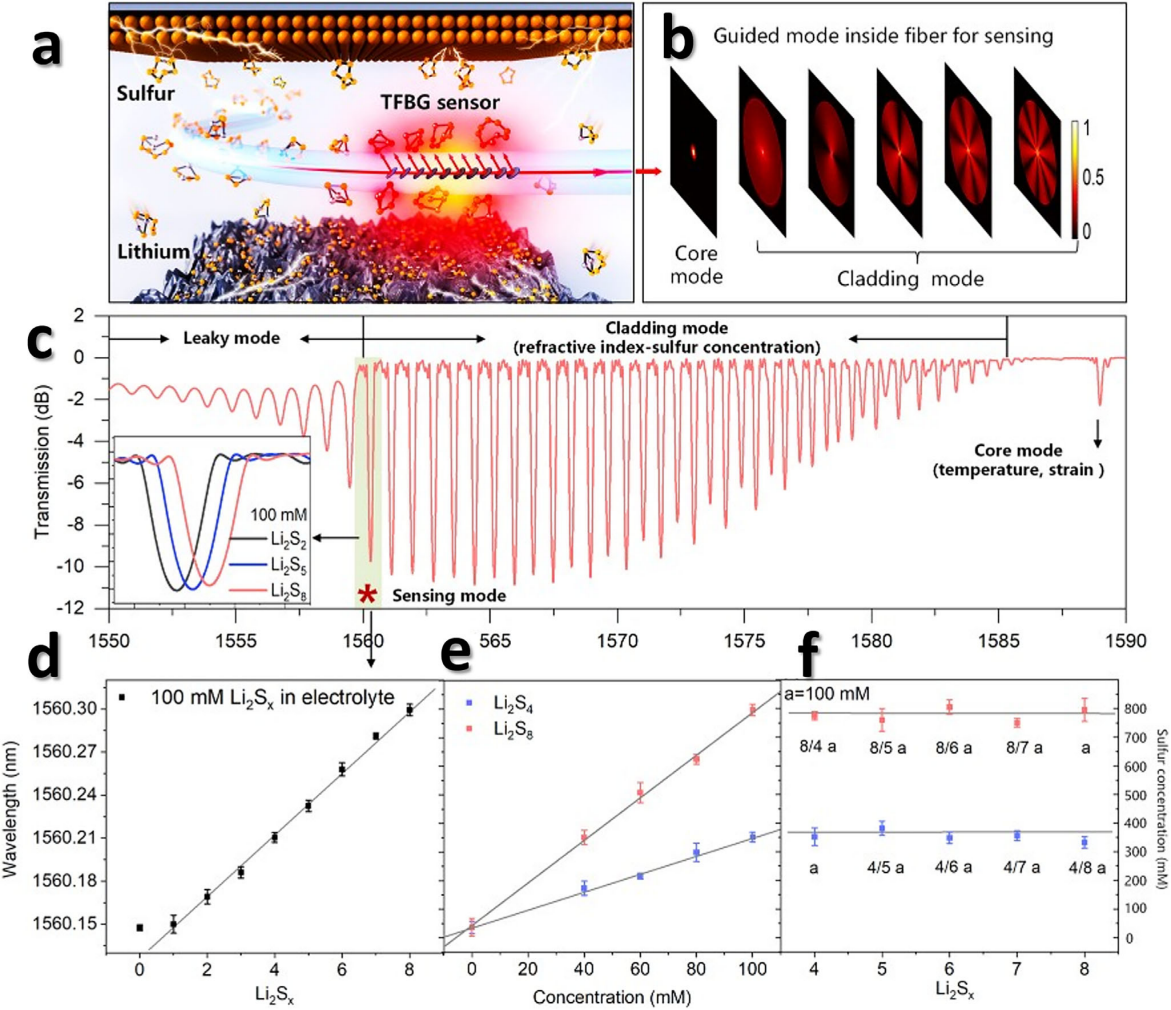
(a) Schematic of a fiber optic sensor immersed in electrolyte for in situ detection of sulfur concentration originating from the generated dissolved polysulfide and their transport activities (i.e., shuttle effect). (b) Backward‐propagation guided modes inside fiber for sensing. (c) Experimental spectra response to polysulfide. (d) The wavelength shifts of cladding mode resonance at ∼1560 nm to 100 mM polysulfide Li_2_S*_x_* (*x*  =  1, 2, 3, …, 8), shaded in green. (e) to concentration variation of Li_2_S_4_ and Li_2_S_8_ from 0 to 100 mM. (f) to same sulfur concentration of polysulfide Li_2_S*_x_* (*x*  =  4, 5, 6, 7, 8). The error bars represent the measurement error (test 3 times continuously) resulting from the surrounding temperature change and electrolyte solvent evaporation [141]. Adapted with permission from Ref. [141]. Copyright 2023, Springer Nature.

Advanced imaging versions of these techniques map processes spatially. Operando confocal Raman microscopy visually tracks the generation, evolution, and diffusion of polysulfides within a working cell, revealing stepwise discharge versus parallel recharge pathways and correlating local polysulfide concentration with performance [139]. The ultimate spatial resolution is achieved by in situ electrochemical transmission electron microscopy (TEM). A landmark study by Zhou et al. used liquid‐cell electrochemical TEM to visualize interfacial conversion at the atomic scale, capturing unexpected collective charge transfer and instantaneous Li_2_S deposition on nanocluster‐active surfaces directly validating that tailored surfaces alter the fundamental reaction mechanism [140]. Emerging optical sensing techniques offer a new dimension of quantitative, real‐time data. Liu et al. pioneered the use of tilted fiber Bragg grating (TFBG) sensors to operando monitor the refractive index and temperature within a Li–S cell, allowing quantitative tracking of sulfur concentration in the electrolyte and revealing how Li_2_S nucleation pathways govern cycling performance [141]. Figure 11a presents a schematic of a fiber optic sensor immersed in electrolyte for in situ detection of sulfur concentration originating from dissolved polysulfides and their transport activities (i.e., the shuttle effect). Figure 11b illustrates the backward‐propagation guided modes inside the optical fiber that are responsible for sensing, where changes in the surrounding refractive index induce measurable shifts in the cladding mode resonances. Figure 11c shows experimental spectra responses to polysulfide exposure, demonstrating clear changes in the resonance features as polysulfide concentration varies. Figure 11d displays the wavelength shifts of cladding mode resonance at approximately 1560 nm in response to 100 mM polysulfide Li_2_S_x_ (where x = 1, 2, 3, …, 8), with the green‐shaded region highlighting the sensitivity range; distinct shift patterns are observed for different polysulfide chain lengths. Figure 11e presents the sensor response to concentration variation of Li_2_S_4_ and Li_2_S_8_ from 0 to 100 mM, showing a linear correlation between wavelength shift and polysulfide concentration. Figure 11f shows the response to the same sulfur concentration of polysulfide Li_2_S_x_ (x = 4, 5, 6, 7, 8), demonstrating that the sensor can distinguish between different polysulfide species. The error bars represent measurement error from three continuous tests, accounting for surrounding temperature changes and electrolyte solvent evaporation.

Beyond electrolyte chemistry, such tools can probe the solid‐electrolyte interface itself; Han et al. used ultrasensitive TFBG sensors to *operando* monitor lithium dendrite growth and mass transport kinetics at the Li metal anode interface, providing critical diagnostics for anode‐side failures that plague practical cells [142].

Characterization is equally vital for resolving the static, nanoscale structure of materials that dictate performance. For instance, Yamaguchi et al. used small‐angle X‐ray scattering and pair distribution function analysis to uncover the local structure of sulfur in carbon‐sulfur composites for all‐solid‐state batteries, revealing that melt‐diffused sulfur is amorphous and consists of S_8_ rings a structure that facilitates extensive carbon‐sulfur contact and leads to near‐theoretical capacity utilization [143].

Recent studies link host structure directly to reaction dynamics; Bowman et al. used *operando* XRD on hollow carbon sphere hosts to reveal that Li_2_S crystallite growth plateaus mid‐discharge due to the formation of an amorphous polysulfide matrix that restricts further crystallization, providing a specific mechanistic explanation for capacity limits [144]. The most comprehensive approach aims to reconstruct entire reaction networks. A seminal 2024 study by Liu et al. [145] combined in situ Raman, electrochemistry, and DFT to map the sulfur reduction reaction (SRR) network, identifying Li_2_S_4_ as the key electrochemical intermediate and Li_2_S_6_ as the primary shuttling species generated via chemical comproportionation. This complete mechanistic framework demonstrates how catalytic hosts accelerate Li_2_S_4_ conversion to suppress shuttle, transforming catalyst design from guesswork into network engineering [145]. To directly probe these complex pathways, Yu et al. pioneered an in situ hyphenated technique combining electrochemistry with mass spectrometry (EC‐MS), enabling real‐time, direct detection of lithium polysulfide species during operation and providing unambiguous chemical proof of catalytic selectivity [146].

Computational modeling, particularly density functional theory (DFT), is essential for screening materials and predicting behavior. DFT can screen polysulfide binding energies on thousands of potential hosts or predict decomposition pathways of electrolyte additives on specific surfaces. However, the accuracy of these predictions hinges on properly describing weak interactions. Fang et al. highlighted that the many‐body dispersion (MBD) method provides a more accurate description of polysulfide anchoring on materials like MXenes and doped graphene compared to simpler van der Waals corrections, revealing that many‐body effects can significantly reduce predicted binding energies a crucial methodological correction for computational screening [147].

These tools are pivotal for uncovering non‐intuitive design principles and establishing fundamental electronic descriptors. For example, DFT elucidated how biaxial strain in 2D catalysts shifts polysulfide adsorption mechanisms and lowers reaction barriers, a principle translated into high‐performance pouch cells [148]. A striking combined theoretical and experimental study by Wu et al. unveiled an autocatalytic growth mechanism of Li_2_S on specific crystal planes of a single‐atom Ni catalyst, where the Li_2_S (100) product surface itself catalyzes further polysulfide conversion shifting the design paradigm toward engineering interfaces that foster catalytically active product layers [149]. Moving from empirical discovery to rational design requires establishing fundamental descriptors. Wang et al. decoded design rules using SmMn_2_O_5_ mullite, identifying lattice matching and 3d‐orbital selection as critical criteria, with specific Mn orbitals near the Fermi level coupling strongly with polysulfide p‐orbitals to reduce overpotentials [150]. Similarly, Hua et al. revealed that the p electron gain of (S) in p‐block metal sulfides linearly correlates with the activation energy for Li_2_S deposition, leading to the selection of Bi_2_S_3_ as an optimal catalyst and enabling monumental areal capacity [151]. Fundamental kinetic studies by Peng et al. directly profiled SRR activation energies, confirming that conversion of polysulfides to Li_2_S_2_/Li_2_S is the kinetically sluggish step and linking improved kinetics to a tuned p‐band center [152].

The pursuit of ultimate catalytic control is evolving toward the coordinated modulation of multiple coupled physical fields. Yu et al. [153] demonstrated a synergistic electric‐spin‐dipole modulation strategy, where a composite architecture of amorphous CoB in Co‐CoP heterojunctions allows precise control over the built‐in electric field, spin states, and dipole interactions. This tripartite synergy creates an optimal environment for bidirectional polysulfide confinement and conversion, providing a comprehensive multi‐dimensional design principle [153]. A robust thermodynamic foundation is essential for predictive models. A breakthrough by Song et al. [154] constructed the first comprehensive ternary phase diagram (S‐Li_2_S‐polysulfides) for Li–S batteries based on phase equilibrium analysis. This work quantitatively explained the discharge profile, identified intrinsic deposition limits, and established the relationship between system state and equilibrium potential, providing an essential framework for validating and parameterizing advanced models [154].

Advanced characterization can directly inform redesign to overcome specific challenges. Kim et al. [155] used XAS and ToF‐SIMS to reveal that the discharge product in all‐solid‐state Li–S batteries is a mixture of Li_2_S and Li_2_S_2_, not pure Li_2_S. Leveraging this insight, they adjusted the discharge cutoff potential and added a catalyst to favor the more reversible Li_2_S_2_ phase, achieving remarkable stability over 1500 cycles [155]. For wide‐temperature operation, Deng et al. [156] used in situ Raman and impedance spectroscopy to identify a temperature‐dependent shift in the rate‐determining step, revealing that at cryogenic temperatures, reduction of Li_2_S_8_ to Li_2_S_4_ becomes critical. With this mechanistic understanding, they designed a host to specifically accelerate these targeted steps, enabling stable operation across a wide temperature range [156]. In summary, advanced characterization and modeling are not merely supportive tools but are central to the modern, rational design of Li–S batteries. By providing atomic‐scale insights into mechanisms, quantifying dynamic processes, and establishing predictive design rules, they enable a transition from trial‐and‐error material discovery to the engineering of optimized, multifunctional systems capable of meeting the stringent demands of practical high‐energy‐density cells.

### 4.3 Introducing AI/ML for Accelerated Discovery

The complex, multi‐parameter optimization required for practical Li–S batteries presents an ideal challenge for Artificial Intelligence and Machine Learning (AI/ML). These techniques can uncover hidden patterns in high‐dimensional data beyond human intuition, acting as a co‐pilot for discovery. The process begins by constructing a unified “Materials Genome” for Li–S, integrating performance data from literature and experiments with synthetic data from density functional theory (DFT) calculations, material descriptors (surface area, porosity, composition), and electrolyte properties (donor number, concentration, viscosity). Graph neural networks can model the relationships within this dataset, learning the hidden rules linking composition and configuration to performance. Once trained, such models enable inverse design: instead of predicting outcomes for a given material, they can propose candidate compositions and cell architectures optimized for specific goals e.g., “maximize areal capacity at 100 cycles with E/S < 3 µL mg^−1^ and loading > 4 mg cm^−2^.” ML can also guide the search for synergistic electrolyte‐cathode pairs by identifying complementary properties. Active learning loops, where model predictions are tested in the lab and results fed back to refine the model, can dramatically compress the discovery timeline, offering a credible path to solving the intertwined challenges of stability, energy density, and rate capability.

**FIGURE 12:**
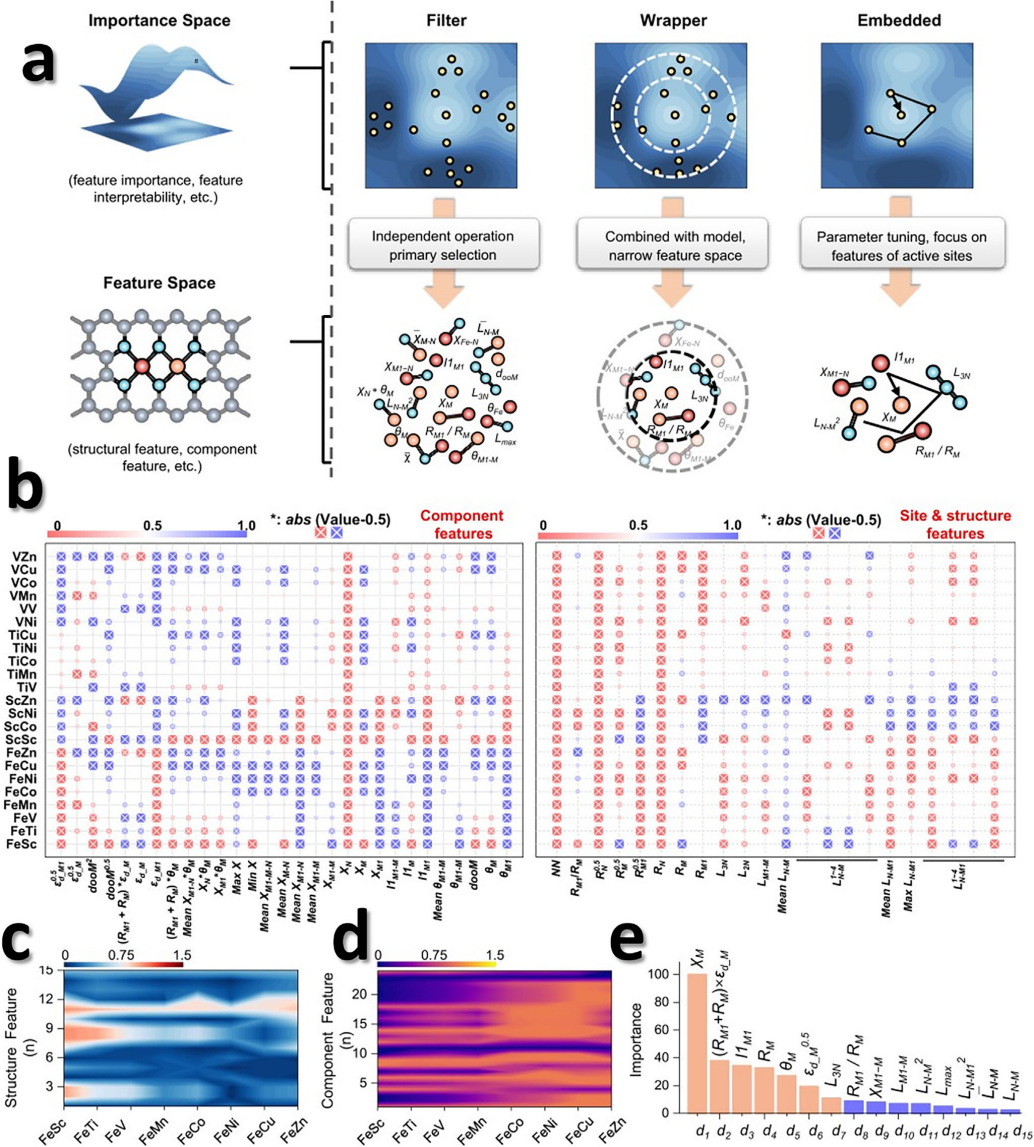
(a) Schematic of the different approaches toward feature engineering. (b) The original dataset represented by the normalized data (only component features and site & structure features are shown. (c,d) Feature heat maps (including structure and component features) of the partial DASC (Main metal  =  Fe). (e) Feature importance (%) of the final XGBR model based on embedded module results [158]. Adapted with permission from Ref. [158]. Copyright 2024, Springer Nature.

Jeschke and Johansson [157] present a pioneering approach to applying supervised machine learning for the classification of Li–S battery electrolytes by predicting polysulfide solubility a priori. The authors highlight that modern battery R&D, particularly for complex systems like Li–S, involves numerous multi‐variable problems where traditional trial‐and‐error methods are inefficient. To address this, they developed a framework that combines density functional theory (DFT) with statistical mechanics (COSMO‐RS) to create a quantitative structure‐property relationship (QSPR) model. This model enables the accurate classification of different electrolyte compositions based on their predicted interaction with polysulfides, a critical factor for managing the shuttle effect [157]. Landmark studies demonstrate this integrated approach. Han et al. [158] developed a multi‐view machine learning framework using intrinsic electronic descriptors to design transition metal electrocatalysts. Guided by the model, they synthesized a carbon‐coated Fe/Co catalyst from recycled materials, which delivered an initial specific energy of 436 Wh kg^−1^ in a high‐loading pouch cell, completing the loop from computational design to practical validation [158]. Figure 12a presents a schematic of the different approaches toward feature engineering in machine learning for catalyst design. Three methods are illustrated: filter methods (independent operation for primary selection), wrapper methods (combined with model to narrow feature space), and embedded methods (parameter tuning focusing on features of active sites). Figure 12b shows the original dataset represented by normalized data, including component features and site and structure features for various transition metal combinations (e.g., VZn, VCu, FeZn, FeCu, FeNi, FeCo, etc.). Figure 12c displays feature heat maps of structure features for the partial dual‐atom single‐carbon (DASC) catalyst dataset with iron as the main metal, where color intensity indicates correlation strength (ranging from 0.75 to 1.5). Figure 12d presents feature heat maps of component features for the same DASC dataset (main metal = Fe), revealing correlations among different elemental and structural descriptors. Figure 12e shows the feature importance (%) of the final XGBoost regression (XGBR) model based on embedded module results, identifying the most critical descriptors (e.g., Fe‐related, Co‐related, Ni‐related, Cu‐related, and Zn‐related features) that govern electrocatalytic activity for sulfur conversion.

Similarly, Lee et al. created an attention‐based ML model to predict the Gibbs free energy of mixing (∆G_mix_) for electrolyte formulations a key determinant of polysulfide solubility enabling rapid screening of vast chemical spaces and providing interpretable insights into molecular features governing performance [159]. Beyond electrolyte screening, machine learning is also being applied to optimize other critical components and their synthesis. Qiu et al. [160] present a concept article focused on the application of machine learning for the design and optimization of multicomponent electrolytes in lithium–sulfur batteries. The authors highlight the critical challenge posed by the complex interactions between multiple electrolyte components and the intricate formation mechanisms of electrode‐electrolyte passivation layers, which are difficult to unravel through conventional trial‐and‐error methods. They position machine learning as a time‐saving and highly efficient tool to accelerate the discovery and optimization of novel electrolyte formulations [160]. Furthermore, ML can extract fundamental design principles. Wang et al. [161] used ML to identify p‐p‐s orbital coupling as a key electronic descriptor for anion‐doped catalysts, establishing a volcano relationship that pinpointed moderate coupling as optimal. This rule guided the synthesis of a B‐doped WSe_2_/MXene catalyst, enabling a high‐energy 3 Ah pouch cell [161]. In another landmark, Han et al. [162] used ML to derive a binary descriptor combining a band match index (I_Band_) and a lattice mismatch index (I_Latt_) for sulfur reduction catalysis. Guided by this descriptor, they identified and synthesized NiSe_2_, which enabled stable operation under high sulfur loading (15 mg cm^−2^), at ‐20°C, and in a 402 Wh kg^−1^ pouch cell [162].

**FIGURE 13:**
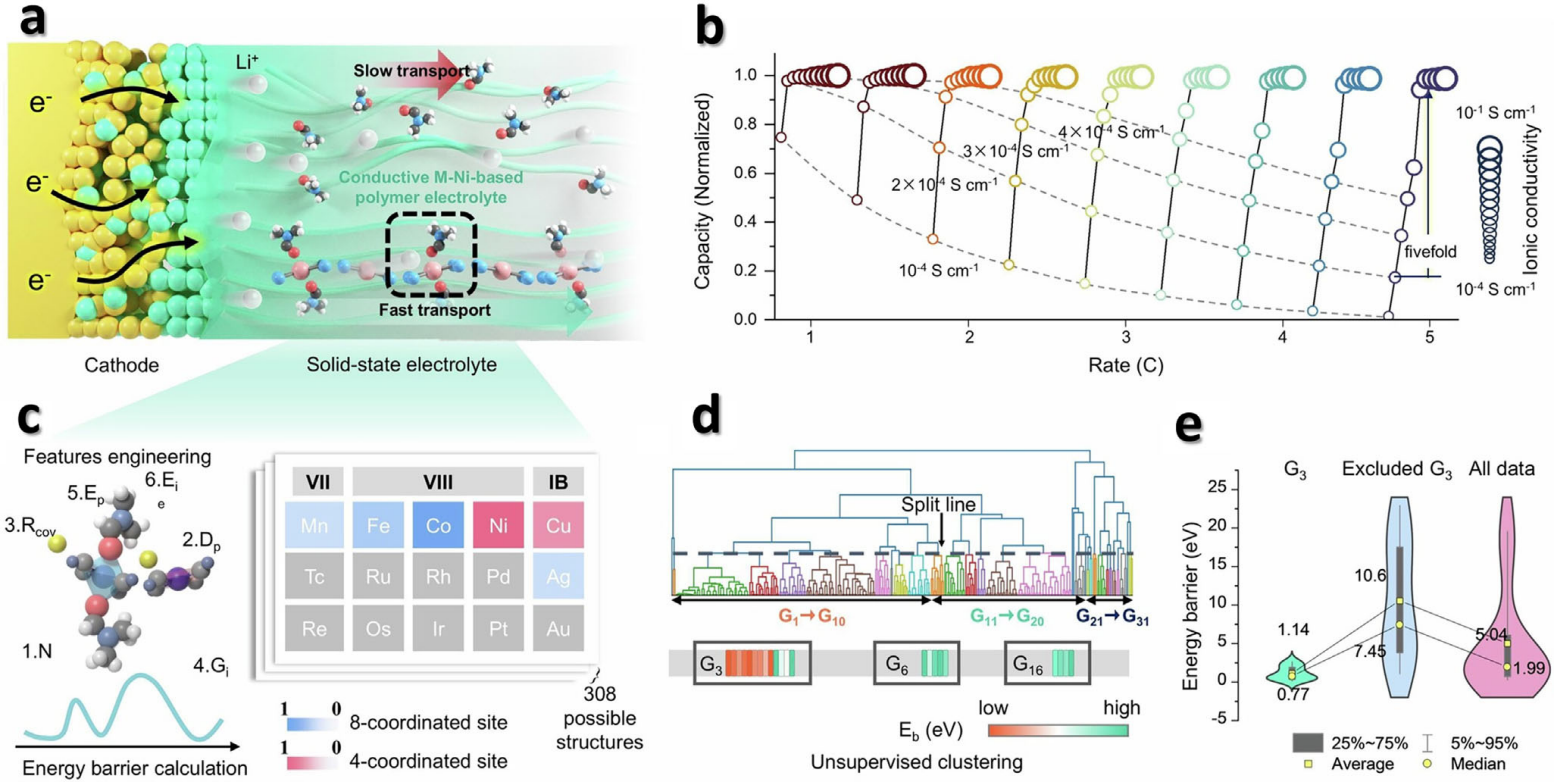
(a) Schematic illustration of polymer electrolyte system and the need of high ionic conductivities. (b) The relationship between battery capacity, rate, and the ionic conductivity of the electrolytes where different colors indicate the charge/discharge rates from 1 C to 5 C and sizes of spheres represent various ionic conductivities of the electrolytes. (c) Construction of sample database and fingerprints constitute model features to predict E_b_ where blue and red elements represent octahedral sites M_1_, and square planar sites M_2_. (d) Bottom‐up tree (dendrogram) generating AHC algorithm where all Hofmann complexes are divided into 31 groups, labeled G_1_‐G_31_, and different groups are denoted in different colors. (e) Violin plots demonstrating E_b_ distribution [164]. Adapted with permission from Ref. [164]. Copyright 2025, Springer Nature.

Machine learning is also proving powerful for optimizing synthesis parameters. Chen et al. [163] present a machine learning‐driven strategy to design MoS_2_/MoO_3_ heterostructures anchored on nitrogen‐doped hollow carbon shells for polysulfide mitigation in lithium–sulfur batteries. The authors employ gradient boosting decision trees (GBDT) modeling to optimize critical synthesis parameters, such as carbonization temperature and oxidation duration, aiming to achieve an optimal balance between adsorption capacity and catalytic activity. This ML‐guided approach successfully identified the conditions for producing heterostructures that exhibit superior polysulfide confinement and accelerated conversion kinetics. The resulting material, when integrated into a modified separator, enabled Li–S coin cells to deliver a remarkable initial capacity of 1002 mAh g^−1^ at 1C with low decay over 500 cycles. Importantly, the authors validated their approach under practical conditions, demonstrating that a pouch cell with high sulfur loading (8.4 mg cm^−2^) maintained an areal capacity of 7.25 mAh cm^−2^ with 90.1% retention after 100 cycles [163]. AI/ML also accelerates the discovery of critical components like solid electrolytes. Lao et al. [164] used unsupervised learning and density functional theory (DFT) to screen Hofmann complexes for weak Li^+^ coordination environments conducive to fast conduction. This computational discovery pipeline led to a Co(dmf)_2_Ni(CN)_4_‐based solid polymer electrolyte, which enabled high‐performance Li‐SPAN cells scaled to a 0.6 Ah pouch cell [164]. Figure 13a presents a schematic illustration of a polymer electrolyte system, highlighting the need for high ionic conductivities to enable fast Li^+^ transport between the cathode and anode. The schematic shows a conductive M‐Ni‐based polymer electrolyte facilitating rapid ion transport. Figure 13b shows the relationship between battery capacity (normalized), charge/discharge rate (from 1C to 5C, indicated by different colors), and the ionic conductivity of the electrolytes (sphere sizes represent different ionic conductivities ranging from 10^−4^ to >10^0^ S cm^−1^). This plot demonstrates that higher ionic conductivity enables higher rate capability and better capacity retention. Figure 13c illustrates the construction of the sample database and fingerprints that constitute model features to predict binding energy (E_b_). Blue and red elements represent octahedral sites (M_1_) and square planar sites (M_2_) in Hofmann complexes, respectively, which are key structural descriptors for screening. Figure 13d presents a bottom‐up tree (dendrogram) generated by the agglomerative hierarchical clustering (AHC) algorithm, where all Hofmann complexes are divided into 31 groups (labeled G_1_–G_31_). Different groups are denoted in different colors, revealing natural clusters of complexes with similar structural and electronic properties. Figure 13e displays violin plots demonstrating the distribution of predicted binding energies (E_b_) across different clusters, allowing rapid identification of promising candidates with optimal Li^+^ coordination environments for high ionic conductivity.

## 5 Interdependence of the Three Frontiers

The path to practical lithium–sulfur batteries is a convergent effort across interdependent frontiers. This review argues that success hinges not on isolated material breakthroughs but on the synergistic integration of scalable electrode engineering, co‐designed electrolyte‐cathode systems, and data‐driven optimization. The three frontiers scale‐up, integrated design, and data‐driven optimization are not parallel tracks but a tightly interconnected web where progress in one catalyzes and necessitates advances in the others. This interdependence can be visualized as a self‐reinforcing cycle of innovation. For instance, the development of a new multifunctional binder with polysulfide‐adsorbing and ion‐conducting properties (an advance in *integrated design*) directly enables the fabrication of thicker, crack‐free electrodes with higher sulfur loading (progress in *scale‐up*) [165]. However, the true efficacy of this binder under practical conditions cannot be assumed; it must be rigorously validated through data‐driven benchmarking in pouch cells, generating performance maps that quantify its impact on cycle life at varying E/S ratios. Conversely, insights from meta‐analysis of large‐scale performance data (a *data‐driven* activity) may reveal that electrodes exceeding a certain density universally fail, pinpointing a mechanical stress limit.

This discovery directly informs the integrated design of more compliant host architectures or graded electrode structures, which in turn enables further scale‐up to higher loadings without fracture. Similarly, a machine learning model trained on electrochemical and materials data might predict that a specific class of MOFs paired with a localized high‐concentration electrolyte would maximize sulfur utilization. This *data‐driven* prediction guides the *integrated design* of a new cathode‐electrolyte pair, whose promise must then be proven by successful *scale‐up* and testing in a multi‐layer pouch cell. The failure to advance on any one frontier acts as a bottleneck: a brilliant host material cannot be utilized without a scalable manufacturing process, and a scalable electrode cannot achieve longevity without a compatible electrolyte system, whose optimal formulation remains hidden without systematic data analysis [3]. Success, therefore, demands coordinated research programs that actively bridge these domains, treating the Li–S battery as a complex system where material, component, and process innovation are continuously informed by and validated against practical, data‐rich benchmarks. Representative examples of such integrated approaches are summarized in Table 3, which compiles recent advances spanning binder engineering, catalytic and structural host design, electrolyte optimization, and scalable electrode architectures, along with their corresponding performance under practical conditions such as high sulfur loading, lean electrolyte operation, and extended cycling stability.

**TABLE 3:** Emerging strategies for integrated Li–S system design.

Strategy category	Specific approach / material	Performance outcome	Temperature range	References
Binder innovation	γ‐ray‐cross‐linked PAM network (I‐PAM)	High‐loading (6.4 mg cm^−2^) stability Lean E/S (3 µL mg^−1^) compatibility 410 Wh kg^−1^ in 1.2 Ah pouch cell	Wide (278‐328 K)	[166]
Host material (Theory)	Defective biphenylene (BPN) sheet	Strong LiPS anchoring (Superior to graphene, MXenes)	—	[167]
Host material	Polypyrrole/Sulfur composite (S/SP/ppy)	Coin cell: 459 mAh g^−1^ after 100 cycles @ 0.5C	—	[168]
Sustainable host	KOH‐activated olive pomace biocarbon (Solid activation: A5AS9)	Lean E/S compatible (5 µL mg^−1^) High‐loading stable (4 mg cm^−2^, 850 mAh g^−1^) High‐rate (5C, 360 mAh g^−1^) & long life (300+ cycles)	—	[169]
Host material	MOF‐74(Ni) framework	0.001% fade/cycle (99.75% retention) over 200 cycles @ 0.5C Moderate rate capability (315 mAh g^−1^ @ 2C)	RT (25 °C)	[170]
Scalable electrode	N, O‐doped wood‐like C/CNT forest (WLC‐CNTs)	Ultra‐high loading (52.4 mg cm^−2^) Lean E/S (6 µL mg^−1^) compatible Enables Li–S full cell (Li hosted in same framework)	RT (25 °C)	[165]
All‐solid‐state design	Mixed ionic‐electronic conductors (MIECs)	High sulfur utilization (>94%) & capacity (>1450 mAh g^−1^) Long life (>1000 cycles) in ASS Li–S batteries	—	[171]
All‐solid‐state cathode	Conductive S9.3I molecular crystal	400 cycles with 87% retention in SSLSB	—	[172]
Catalytic host	CoZn clusters/C nanocomposite	1000 cycles @ 8C (13.4 A g_s_^−1^) High loading (5 mg cm^−^^2^), lean E/S (4.8) compatible	RT (25 °C)	[173]
Electrolyte additive	Closo‐complex hydride Li(CB_11_H_12_)	Suppresses LiPS dissolution & stabilizes Li anode	RT (25 °C)	[174]

### 5.1 Roadmap for Practical Li–S Batteries

**FIGURE 14:**
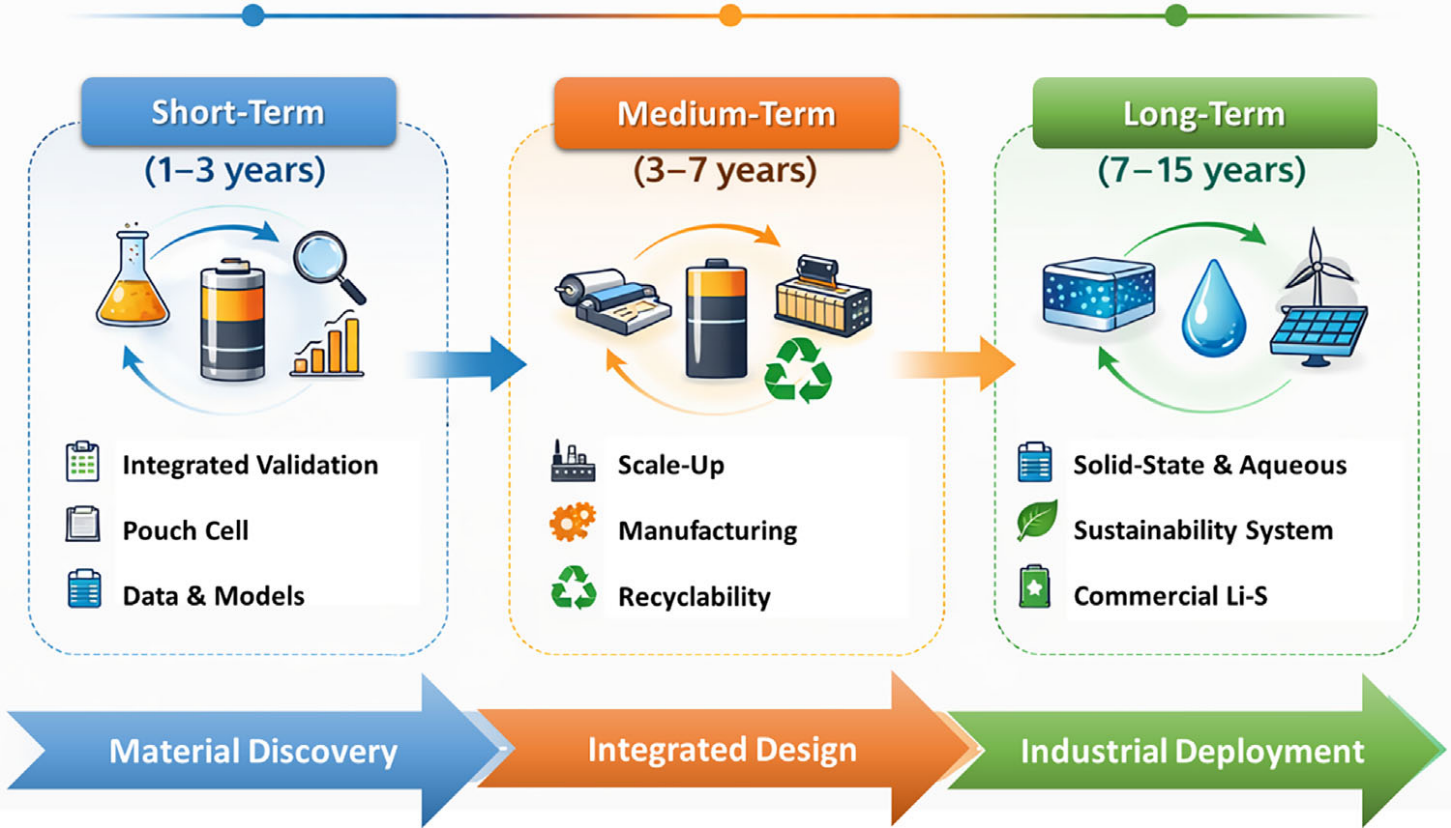
Staged roadmap for the development of practical lithium–sulfur batteries. The short‐term phase (1–3 years) focuses on integrated validation using pouch cells and data‐driven modeling to establish performance benchmarks. The medium‐term phase (3–7 years) addresses scale‐up, manufacturing processes, and recyclability to enable commercial production. The long‐term phase (7–15 years) targets sustainable disruptive systems including solid‐state and aqueous electrolytes, leading to full commercial deployment. This interconnected framework emphasizes that material discovery, integrated design, and industrial deployment must advance synergistically. Original synthesis by authors.

Guided by the principle of integration, a pragmatic roadmap for lithium–sulfur batteries can be charted with staged priorities, each building upon the last to transition from laboratory validation to commercial deployment. This roadmap is visualized schematically in Figure 14, which summarizes the key milestones and focus areas for each phase.

In the Short‐Term (1–3 years), the focus must be on Integrated Validation. The priority should shift decisively from publishing novel nanostructures tested under forgiving, flooded conditions to the rigorous co‐optimization of existing best‐in‐class materials such as polar/catalytic hosts, localized high‐concentration electrolytes (LHCEs), and functional binders. This work must be conducted under strictly practical metrics, including sulfur loadings greater than 4 mg cm^−2^ and electrolyte‐to‐sulfur (E/S) ratios below 3 µL mg^−1^. The goal should be to produce and publicly share high‐quality, standardized data from multilayer pouch cells. Concurrently, efforts must intensify to build open‐access databases of Li–S materials and electrochemical performance, and to develop multi‐physics models that are validated against *operando* characterization data. This foundational work will create the reliable, comparable knowledge base needed for systematic advancement [10, 175, 176].

The Medium‐Term (3–7 years) must expand the focus to Manufacturing and System Integration. With promising material systems identified, research must address scalability and production. This includes developing solvent‐free or aqueous electrode processing routes, roll‐to‐roll compatible fabrication of free‐standing electrodes or coated interlayers, and designing explicitly for recyclability from the outset. Innovative fabrication techniques are emerging to bridge the lab‐to‐fab gap; for instance, Yang et al. demonstrated a single‐step laser printing strategy that simultaneously synthesizes halloysite‐based hybrid host materials, sulfur, and conductive carbon, depositing them as a uniform composite cathode and bypassing multiple traditional processing steps [28]. Promisingly, alternative cell configurations that circumvent the challenging lithium metal anode are also emerging. As highlighted by Zhang, the use of pre‐lithiated graphite anodes paired with sulfurized polyacrylonitrile (SPAN) cathodes has enabled Ah‐level pouch cells to achieve over 1000 cycles, representing a pragmatic shift toward safer and more manufacturable systems [177].

**FIGURE 15:**
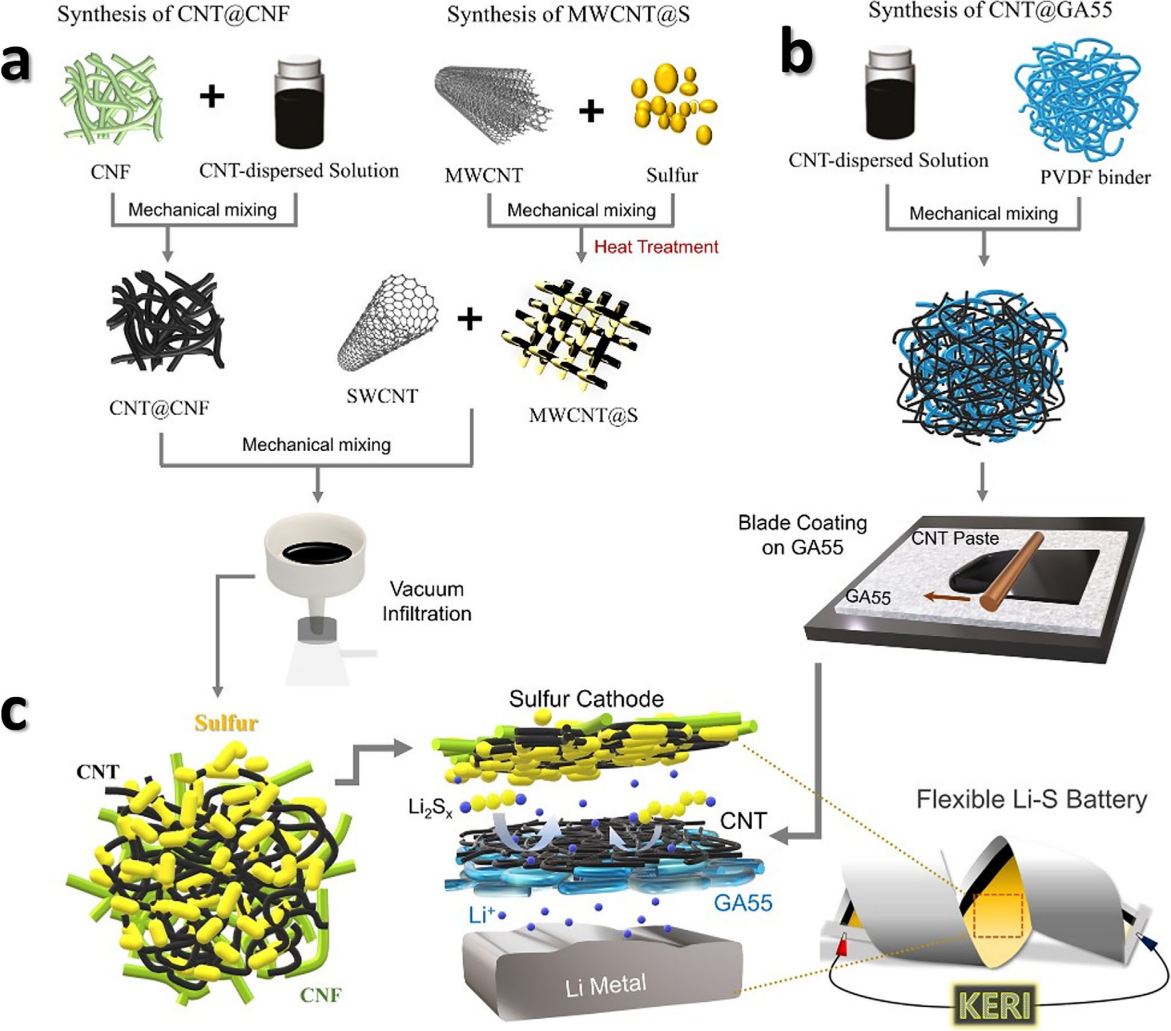
(a) Fabrication processes of CNT@CNF, synthesis of the MWCNT@S powders, and stepwise fabrication flow of the flexible sulfur cathodes. (b) Schematics showing the preparation and fabrication method of the functional carbon nanotube (CNT)‐coated GA55 separator. This component material advantageously mitigates the shuttle effect of lithium polysulfides (Li_2_S_x_). (c) Schematics of the structure of the flexible Li–S battery, wherein the cell is structurally unique with high mechanical flexibility [25]. Adapted with permission from Ref. [25]. Copyright 2021, Springer Nature.

Research must also address application‐specific form factors, such as flexible batteries for wearable electronics, exemplified by the all‐fibrous, flexible Li–S cell achieving 443 Wh kg^−1^ developed by Park et al. [25]. Figure 15a illustrates the fabrication processes of the flexible sulfur cathode, including the synthesis of multi‐walled carbon nanotube@S (MWCNT@S) powders and the stepwise fabrication flow for producing the flexible carbon nanofiber (CNF)‐based cathode. Figure 15b presents schematics showing the preparation and fabrication method of the functional carbon nanotube (CNT)‐coated GA55 separator, which mitigates the polysulfide shuttle effect by providing a conductive and adsorptive barrier. Figure 15c shows the structure of the flexible Li–S battery, wherein the cell is designed with high mechanical flexibility, enabling it to withstand bending and deformation while maintaining electrochemical performance. Early demonstrations of integrated full cells with minimal lithium excess set important precedents. For instance, [178] constructed a flexible full cell using metallic‐coated carbon fabrics as hosts for both sulfur and lithium. This co‐engineering approach stabilized both electrodes, enabling a full cell with only 100% oversized lithium (N/P ≈ 2), a high areal capacity of 3 mAh cm^−2^, and robust cycling a significant step towards practical cell balancing and flexible form factors [178].

A critical and often underrepresented pillar of this stage is the rigorous quantification of environmental impact. Pioneering work by Huang et al. establishes a vital framework by conducting comprehensive life cycle assessments (LCA) of biopolymer‐based components, providing the data‐driven methodology needed to benchmark “green” materials and integrate circularity indices into Li–S design [179]. System‐level integration work remains crucial: engineering stable lithium metal anodes (or their alternatives) specifically for the Li–S chemistry, and designing intelligent battery management systems capable of accounting for Li–S‐specific states like polysulfide shuttle and electrolyte depletion.

The Long‐Term vision (7–15 years) focuses on achieving full technological maturity through Sustainable, Disruptive Systems. This stage involves the exploration of fundamental paradigm shifts. Truly disruptive system redesigns that alter the core stability paradigm are emerging, such as the quasi‐solid aqueous electrolytes (QAEs) developed by Yi et al. [180]. By confining water molecules, this approach enables a Li||S battery with over 4000 cycles, decoupling development from flammable organic solvents and directly addressing sustainability and cost benchmarks for large‐scale storage [180]. The exploration of solid‐state Li–S batteries will be paramount, requiring not only engineered cathode composites but also enabling electrolyte technologies. Parallel to revolutionary “all‐in‐one” designs like the potential‐gated polymer by Xu et al. [181], efforts focus on creating superionic solid electrolytes with tailored functionality, such as the bifunctional polymer (PS‐3H4S) by Wang et al. designed for high conductivity and mechanical strength [182]. To validate progress along this roadmap, we propose a set of specific, measurable milestones for each development phase, as summarized in Table 4, covering key metrics such as energy density, cycle life, sulfur loading, electrolyte consumption, and system‐level targets required for commercialization.

**TABLE 4:** Specific, measurable milestones for validating the integrated design roadmap toward commercialization.

Phase	Milestone	Target value	References
Short‐Term (1–3 years)	Pouch cell energy density	≥400 Wh kg^−1^	[183]
	Cycle life	≥300 cycles at 80% retention	[184]
	Sulfur loading	≥5 mg cm^−2^	[184]
	E/S ratio	≤3 µL mg^−1^	[107]
	Areal capacity	≥5 mAh cm^−2^	[185]
Medium‐Term (3–7 years)	Pouch cell energy density	≥500 Wh kg^−1^	[186]
	Cycle life	≥800 cycles at 80% retention	[186]
	Sulfur loading	≥8 mg cm^−2^	[186]
	E/S ratio	≤2 µL mg^−1^	[187]
	N/P ratio	≤2	[188]
	Manufacturing cost	≤$100 kWh^−1^	[189]
Long‐Term (7–15 years)	Pouch cell energy density	≥600 Wh kg^−1^	[190]
	Cycle life	≥1500 cycles at 80% retention	[191]
	Sustainability	≥90% recyclability	[192]

Safety considerations are equally critical and must be quantitatively integrated into the roadmap. In the short‐term, baseline safety metrics such as open circuit voltage stability and absence of internal short circuits during initial cycling should be established. Medium‐term milestones include demonstrating dendrite‐free lithium plating for over 500 cycles at practical current densities (≥3 mA cm^−2^) and quantifying gas evolution rates below 0.1 mmol g^−1^ over extended cycling. Long‐term targets require passing all UN38.3 safety tests including nail penetration, overcharge, and thermal abuse at the pouch cell level, with particular emphasis on mitigating dendrite‐induced short circuits and managing gas evolution under extreme conditions. These quantitative safety benchmarks are essential for validating commercial viability alongside energy density and cycle life.

### 5.2 Conclusion

The journey of lithium–sulfur batteries from a laboratory curiosity to a cornerstone of future energy storage is entering its most critical phase. This review has systematically charted a path forward, arguing that the era of incremental, single‐component improvements is giving way to the necessity for holistic, system‐level engineering. The interdependent challenges of scalable fabrication, electrochemical synergy, and multi‐parameter optimization are not isolated hurdles but facets of a single, complex engineering problem. Success hinges on recognizing that a brilliant catalytic host is futile without a scalable manufacturing process, a lean electrolyte formulation is ineffective without a compatible cathode architecture, and optimal designs remain hidden without systematic, data‐rich validation. The proposed roadmap moving from Integrated Validation to Manufacturing and System Integration, and ultimately toward Sustainable, Disruptive Systems provides a structured framework for this endeavor. It calls for a concerted community effort to prioritize practical pouch‐cell metrics, build open‐access knowledge bases, and embrace data‐driven discovery tools. By treating the Li–S battery as an integrated system where progress in material science, electrochemistry, and engineering inform and validate one another, researchers can transform the enduring promise of lithium–sulfur chemistry into a commercial reality, unlocking a new echelon of energy density essential for a decarbonized world.

## Conflicts of Interest

The authors declare no conflict of interest

## Data Availability

The data that support the findings of this study are available from the corresponding author upon reasonable request.
